# Cuproptosis and Mitophagy Mediated by the THUMPD1/IGF2R‐Dependent Suppression of AKT and Activation of AMPK Signaling Suppress Lung Adenocarcinoma Progression

**DOI:** 10.1002/advs.202521238

**Published:** 2026-05-04

**Authors:** Kai Wu, Bo Qin, Zhuoyu Gu, Weizheng Ding, Xiaoming Chen, Kaishang Zhang, Yujin Qiao, Shuang Yuan, Song Zhao, Xiangnan Li, Peng Zhang

**Affiliations:** ^1^ Department of Thoracic Surgery the First Affiliated Hospital of Zhengzhou University Zhengzhou Henan Province China; ^2^ Henan Medical Key Laboratory of Thoracic Oncology Zhengzhou Henan Province China; ^3^ Department of Translational Medicine Center the First Affiliated Hospital of Zhengzhou University Zhengzhou Henan Province China; ^4^ Henan Province Engineering Research Center of Molecular Pathology and Clinical Experiment of Thoracic Diseases Zhengzhou Henan Province China

**Keywords:** cuproptosis, IGF2R, lung adenocarcinoma, mitophagy, THUMPD1

## Abstract

Lung adenocarcinoma (LUAD) remains a leading cause of cancer‐related mortality. N4‐acetylcytidine (ac4C) modification regulates mRNA stability and translation, but the role of its associated co‐factor, THUMP domain‐containing protein 1 (THUMPD1), in cancer is unknown. Clinical LUAD samples and Gene Expression Omnibus (GEO) datasets were analyzed for THUMPD1 expression and prognosis. In vitro and in vivo functional assays were performed to assess the impact of THUMPD1 on LUAD. Multi‐omics approaches and mechanistic studies were employed to identify downstream targets and signaling pathways. THUMPD1 was significantly downregulated in advanced‐stage LUAD. THUMPD1 acted as a tumor suppressor, inhibiting LUAD cell proliferation, metastasis, and tumor growth in mouse models. Mechanistically, THUMPD1 directly bound to and upregulated the translation of insulin‐like growth factor 2 receptor (IGF2R) mRNA in an ac4C‐independent manner by facilitating its cytoplasmic localization. The THUMPD1‐IGF2R axis inhibited AKT signaling, which in turn led to the activation of AMPK. This resulted in intracellular Cu^+^ accumulation, triggering cuproptosis and excessive mitophagy, ultimately suppressing tumor growth. Therapeutically, the copper ionophore elesclomol potently inhibited tumor growth in a *Thumpd1*‐knockout mouse model of LUAD.

## Graphical abstract

THUMPD1 drives a tumor‐suppressive signaling cascade in lung adenocarcinoma by promoting IGF2R expression. IGF2R associates with PPP2R1A to suppress AKT and activate AMPK, leading to SLC31A1 upregulation and copper accumulation. Elevated copper disrupts mitochondrial metabolism and induces excessive mitophagy, thereby restraining tumor growth and metastatic progression.

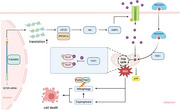

## 1 Introduction

Lung adenocarcinoma (LUAD) is the most prevalent histological subtype of non‐small cell lung cancer and a leading cause of cancer‐related mortality worldwide [1]. According to the Tumor‐Node‐Metastasis (TNM) staging system defined by the American Joint Committee on Cancer (AJCC), LUAD prognosis is closely correlated with disease stage at diagnosis [2]. Early‐stage tumors (stage I‐II) are often amenable to curative surgical resection, whereas advanced‐stage disease (stage III‐IV) with lymph node metastasis or distant organ involvement is associated with significantly poorer outcome [2, 3]. Despite advances in targeted and immunotherapies, the prognosis for patients with advanced or metastatic disease remains poor, underscoring the critical need to identify novel prognostic biomarkers and elucidate the underlying molecular mechanisms driving LUAD pathogenesis [4, 5, 6].

N4‐acetylcytidine (ac4C) is an evolutionarily conserved RNA modification widely present in tRNAs, rRNAs, and mRNAs across prokaryotic and eukaryotic organisms [7]. As the only currently known “writer” of ac4C, N‐acetyltransferase 10 (NAT10) catalyzes ac4C deposition by acetylating cytidine residues, thereby altering the chemical and structural properties of RNA and consequently regulating both target mRNA stability and translation efficiency [8]. Thus, NAT10‐mediated ac4C modification is primarily involved in post‐transcriptional regulation rather than direct transcriptional control. To date, accumulating evidence has shown that NAT10 and its associated ac4C modification are aberrantly upregulated in a variety of malignancies, predominantly in solid tumors, including bladder cancer, hepatocellular carcinoma, cervical cancer, esophageal cancer, and multiple myeloma, where they contribute to tumor cell proliferation, metastasis, drug resistance, and immune evasion [9, 10, 11, 12, 13]. Collectively, these studies support a predominantly pro‐tumorigenic role for NAT10/ac4C in cancer, although the functional consequences of ac4C remodeling may still depend on cellular context and downstream targets.

THUMP domain‐containing protein 1 (THUMPD1) is known to function as an essential activator and co‐factor of NAT10 in the establishment of ac4C modifications on tRNA [8]. However, whether THUMPD1 participates in ac4C deposition on mRNA remains controversial. Schwartz et al. reported that THUMPD1, in conjunction with NAT10, could mediate ac4C modification on mRNA [14]. In contrast, Oberdoerffer et al. found that NAT10‐mediated mRNA ac4C modification occurred independently of THUMPD1, seemingly contradicting earlier findings [7, 14]. Given the generally low abundance of ac4C in mRNA and ongoing advancements in detection methodologies, the role of THUMPD1 in mRNA acetylation remains an open question requiring further investigation [15, 16, 17].

Beyond its molecular function, THUMPD1 plays critical physiological roles. Biallelic variants in THUMPD1 have been linked to syndromic intellectual disability in humans, resulting from loss of tRNA acetylation [18, 19, 20]. In mice, Thumpd1 deficiency has been reported to cause reduced body size and infertility, although adult knockout mice remain viable, while concurrent deletion of Thumpd1 and the stress‐sensing kinase Gcn2 results in penetrant postnatal lethality, together supporting an important role for THUMPD1 in growth, reproductive fitness, and stress adaptation [21]. In cancer, the role of THUMPD1 has been much less explored compared to NAT10. The limited evidence available suggests that THUMPD1 may function in a context‐dependent manner. For instance, THUMPD1 has been implicated in promoting malignant behavior and chemoresistance in gastric cancer, whereas pan‐cancer analyses indicate that its prognostic significance may vary across tumor types [22, 23]. This context‐dependent functionality may stem from the fact that THUMPD1's relationship with NAT10 is not constitutive, but rather substrate‐ and context‐specific. Indeed, whether THUMPD1 is universally required in all biological settings where NAT10 is expressed, or whether its role is restricted to specific RNA substrates and cellular contexts, remains incompletely defined. Likewise, whether THUMPD1 is consistently coexpressed with NAT10 in cancer has not been clearly established.

In LUAD specifically, whether THUMPD1 also regulates ac4C deposition on mRNA, and whether it exerts functions beyond canonical ac4C‐dependent regulation remains largely unknown. In this study, we first identified that THUMPD1 is downregulated in advanced‐stage LUAD and then investigated its potential roles in mRNA ac4C modification and translation regulation. Our findings further indicated that it also regulates key cancer hallmarks, including proliferation, metastasis, and novel cell death pathways such as cuproptosis and mitophagy, through mechanisms independent of ac4C.

## 2 Results

### 2.1 Low Expression of THUMPD1 is Associated With Advanced Disease and Poor Prognosis in LUAD

**FIGURE 1:**
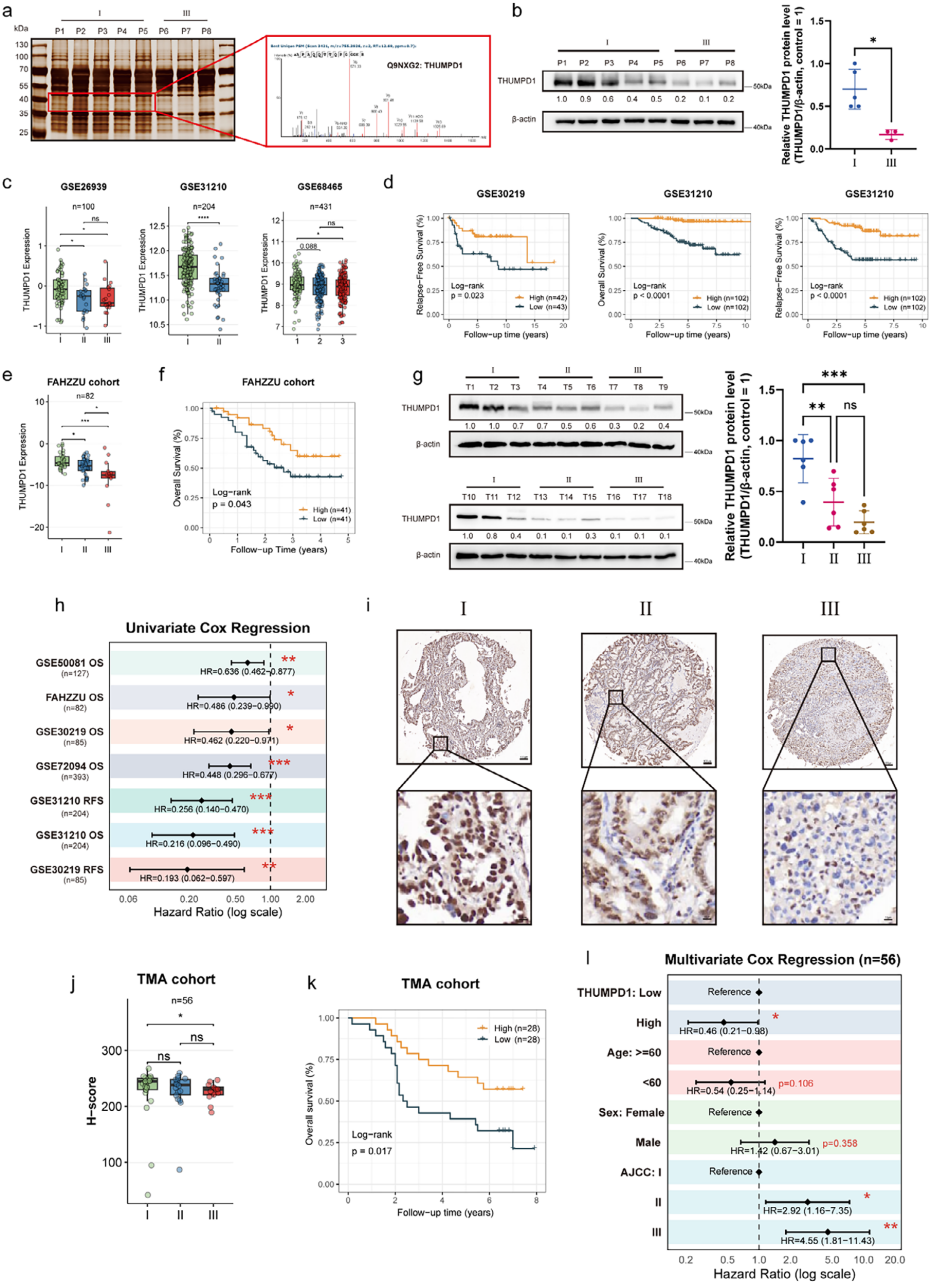
Discovery and clinical association of THUMPD1 in LUAD. (a) Silver‐stained SDS–PAGE of proteins from LUAD tissues stratified by AJCC stage I and III (left); the ∼40‐kDa band (red rectangle) was excised for LC–MS/MS, which identified THUMPD1 (UniProt Q9NXG2; representative spectrum shown, right). (b) Western blot of THUMPD1 in stage I versus stage III LUAD tissues (left, n = 8); quantitative densitometry of THUMPD1 protein levels (right). (c) Boxplots showing THUMPD1 mRNA expression across AJCC stages in external GEO cohorts (GSE26939, n = 100; GSE31210, n = 204; GSE68465, n = 431). (d) Kaplan–Meier curves for overall survival (OS) and recurrence‐free survival (RFS) in GEO cohorts (GSE30219 RFS, n = 85; GSE31210 OS and RFS, n = 204) stratified by THUMPD1 high versus low expression. (e) Boxplot showing THUMPD1 mRNA expression by stage (I–III) in the FAHZZU cohort (n = 82). (f) Kaplan–Meier curve showing OS in the FAHZZU cohort grouped by THUMPD1 high versus low expression (n = 82). (g) Western blot of THUMPD1 in primary LUAD tumors across stages I‐III (left; n = 18); quantitative analysis of THUMPD1 protein expression by stage (right). (h) Forest plot of univariate Cox regression analysis for THUMPD1 across independent datasets (GSE50081 OS, n = 127; FAHZZU OS, n = 82; GSE30219 OS/RFS, n = 85; GSE72094 OS, n = 393; GSE31210 RFS/OS, n = 204). (i) Representative immunohistochemistry (IHC) staining for THUMPD1 in LUAD tissue microarrays (TMAs) across stages I–III. j) Boxplot showing H‐score quantification of THUMPD1 IHC staining in TMA across AJCC stages I‐III (n = 56). (k) Kaplan‐Meier survival analysis in the TMA cohort stratified by THUMPD1 expression (high vs low, n = 56). (l) Multivariable Cox regression in the TMA cohort (n = 56). Values are mean ± s.d. in b and g. Two‐tailed Student's t‐tests were used in b. One‐way ANOVA was used in g. Kruskal–Wallis tests were used in c, e and j, except for GSE31210 in c, in which two stages (I and II) were compared using a Wilcoxon test. Log‐rank tests were used in d, f and k. Univariate and multivariable Cox proportional hazards regression models were used in h and l, respectively. Significance: *p < 0.05; **p < 0.01; ***p < 0.001; ****p < 0.0001; ns, not significant. Original unprocessed blots are shown in Figure S11.

Since AJCC stage significantly influences LUAD prognosis, we began by extracting proteins from 5 stage I and 3 stage III tissues to identify key regulators. A protein band visualized with silver staining around 40 kDa showed higher expression in stage I (Figure 1a, left). The excised bands were analyzed by liquid chromatography‐tandem mass spectrometry (LC‐MS/MS), leading to the identification of THUMPD1 (Q9NXG2) (Figure 1a, right). Western blot confirmed the downregulation of THUMPD1 in stage III tissues (Figure 1b). Consistently, analysis of independent cohorts demonstrated that THUMPD1 expression was negatively associated with advanced AJCC stage (Figure 1c). Kaplan–Meier survival analysis revealed that patients with high THUMPD1 expression exhibited significantly better overall survival (Figure 1d; Figure S1a,b). Similar results were observed in the First Affiliated Hospital of Zhengzhou University (FAHZZU) cohort (Figure 1e‐f).

To further validate these findings at the protein level, we analyzed an additional set of 18 LUAD specimens and observed reduced THUMPD1 expression in advanced‐stage tumors (Figure 1g). Univariate Cox regression analysis across these cohorts indicated that THUMPD1 expression was significantly associated with improved prognosis (Figure 1h). Tissue microarray (TMA) cohort indicated that THUMPD1 is primarily localized in the nucleus and is more highly expressed in early‐stage tumors (Figure 1i,j). Kaplan–Meier survival analysis (Figure 1k) and multivariate Cox regression (Figure 1l) further demonstrated that high THUMPD1 expression predicts better prognosis. Finally, to further strengthen these findings, additional multivariate Cox analyses were performed in independent cohorts, which consistently supported THUMPD1 as an independent prognostic factor in LUAD (Figure S1c–g).

Together, these findings indicate that THUMPD1 is downregulated in advanced LUAD and serves as a favorable prognostic factor.

### 2.2 THUMPD1 Inhibits the Proliferation, Migration and Invasion of LUAD

**FIGURE 2:**
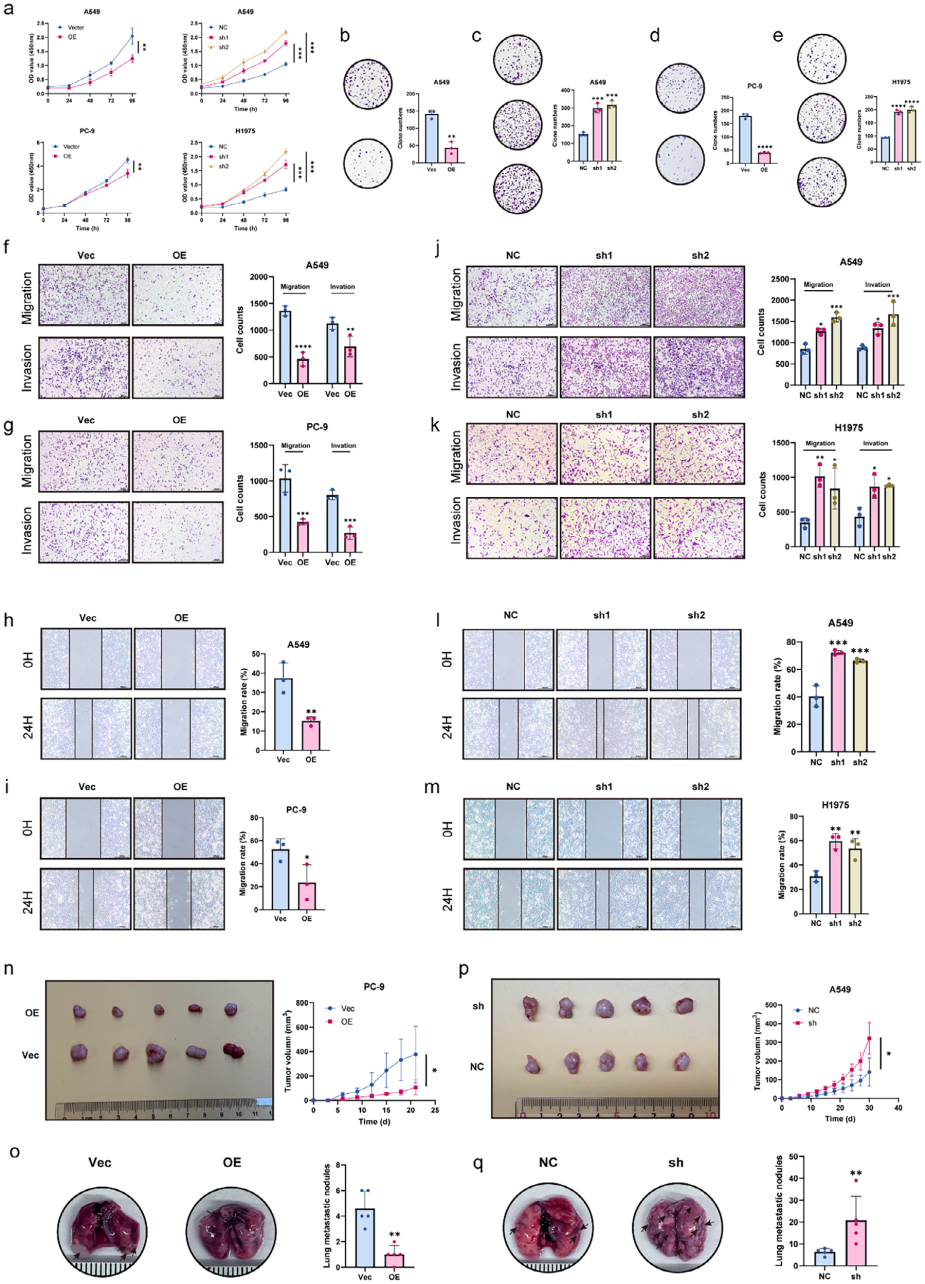
THUMPD1 suppresses LUAD cell proliferation, migration, and invasion in vitro and in vivo. (a) CCK‐8 assays showing the effects of THUMPD1 overexpression or knockdown in A549, PC‐9, and H1975 cells, as indicated. (b–e) Representative images and quantification of colony‐formation assays in A549 cells with THUMPD1 overexpression (b) or knockdown (c), PC‐9 cells with THUMPD1 overexpression (d), and H1975 cells with THUMPD1 knockdown (e). (f–g) Transwell migration and invasion assays with representative fields and quantification in A549 (f) and PC‐9 (g) cells with THUMPD1 overexpression. (h–i) Wound‐healing assays at 0 h and 24 h with migration‐rate quantification in A549 (h) and PC‐9 (i) cells with THUMPD1 overexpression. (j–k) Transwell migration and invasion assays with representative fields and quantification in A549 (j) and H1975 (k) cells with THUMPD1 knockdown. (l–m) Wound‐healing assays at 0 h and 24 h with migration‐rate quantification in A549 (l) and H1975 (m) cells with THUMPD1 knockdown. (n–o) Subcutaneous xenograft and experimental lung metastasis assays using PC‐9 cells with THUMPD1 overexpression, including tumor image, tumor‐growth curves, and quantification of pulmonary metastatic nodules. (p–q) Subcutaneous xenograft and experimental lung metastasis assays using A549 cells with THUMPD1 overexpression, including tumor image, tumor‐growth curves, and quantification of pulmonary metastatic nodules. Values are mean ± s.d. of n = 3 biological replicates in a–m. Xenograft and metastasis experiments were performed with n = 5 mice per group in n–q. Two‐tailed Student's t‐tests were used for comparisons between two groups, and one‐way ANOVA was used for comparisons among multiple groups, as appropriate. Significance: *p < 0.05; **p < 0.01; ***p < 0.001; ****p < 0.0001.

To investigate the phenotypic role of THUMPD1 in LUAD, we used a lentiviral system to generate stable THUMPD1‐knockdown (sh1, sh2) and overexpression (OE) lines in A549, PC‐9, and H1975 cells. These three LUAD cell lines were selected to capture distinct genetic and signaling contexts, thereby improving the generalizability of our findings. A549 represents an EGFR–wild‐type, KRAS‐mutant model, whereas PC‐9 (EGFR exon 19 deletion) and H1975 (EGFR L858R/T790M) are well‐established EGFR‐mutant LUAD models with different EGFR‐driven signaling states. Transduction efficiency in all three cell lines was confirmed by qRT‐PCR and Western blot (Figure S2a–d). CCK‐8 and colony‐formation assays showed that THUMPD1 overexpression suppressed proliferation and viability, whereas THUMPD1 knockdown produced the opposite effects (Figure 2a–e). In addition, transwell and wound‐healing assays indicated that THUMPD1 overexpression attenuated both migration and invasion (Figure 2f–i), while THUMPD1 knockdown enhanced these capacities (Figure 2j–m).

We further examined the effects of THUMPD1 in vivo. Stable cell lines were injected subcutaneously into nude mice to establish xenograft models and metastasis models were generated by tail‐vein injection in the same strain. THUMPD1 overexpression reduced tumor growth, tumor weight, and the number of lung metastatic nodules (Figure 2n,o; Figure S2e,f), whereas THUMPD1 knockdown increased tumor volume and weight and promoted pulmonary metastatic colonization (Figure 2p,q; Figure S2g,h). Collectively, these in vitro and in vivo findings substantiate a tumor‐suppressive role for THUMPD1 in LUAD.

### 2.3 THUMPD1 Potentially Regulates mRNA ac4C Modification and Translation

**FIGURE 3:**
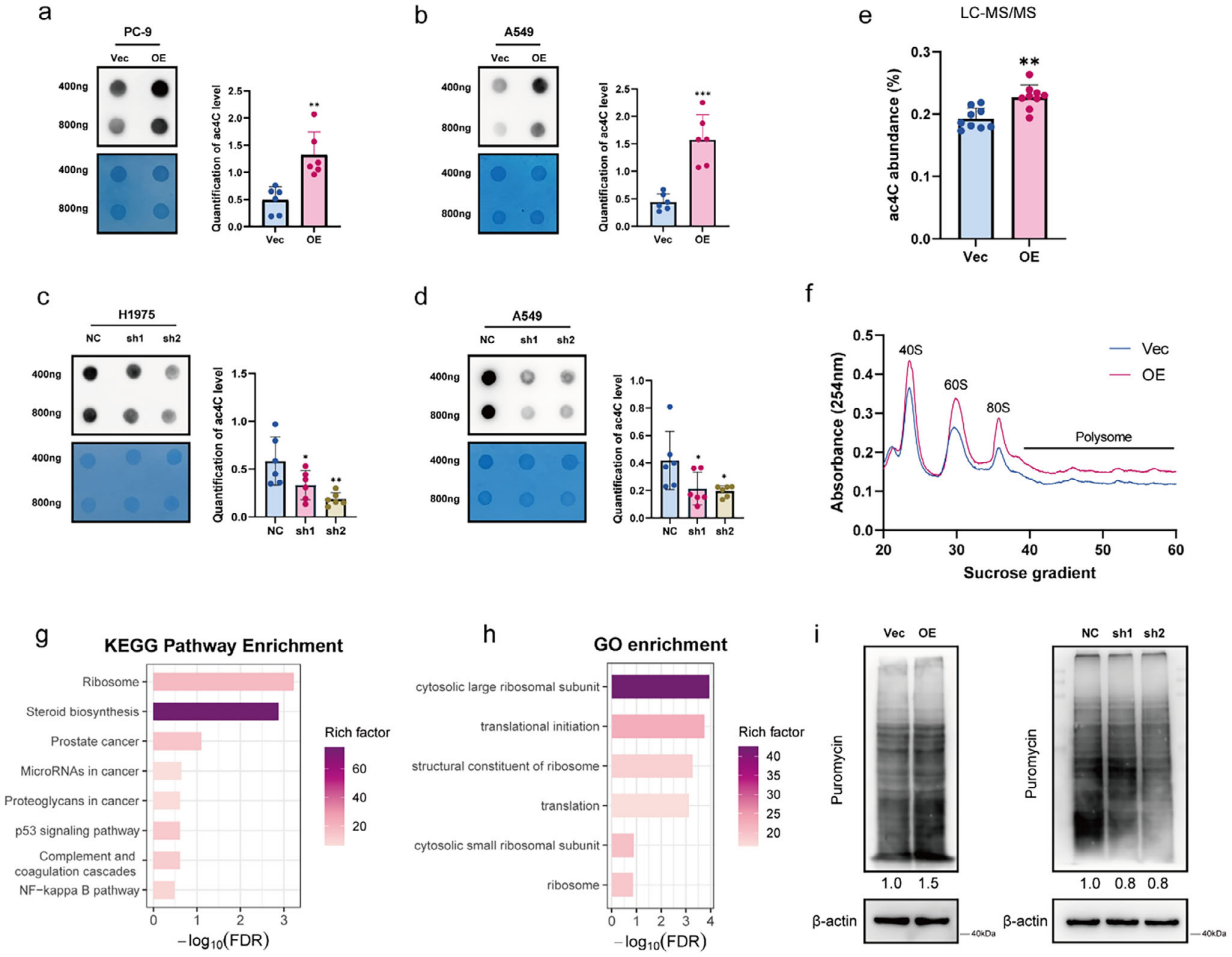
THUMPD1 potentially regulates mRNA ac4C and promotes translation. (a–d) ac4C dot blot assays in PC‑9 (a) and A549 (b) cells with THUMPD1 overexpression, and in H1975 (c) and A549 (d) cells with THUMPD1 knockdown; methylene blue (MB) staining shows equal RNA loading; right, quantification of ac4C levels. (e) LC–MS/MS quantification of global ac4C abundance in Vec vs OE cells. (f) Polysome profiling showed increased polysome fractions in THUMPD1‐overexpressing cells compared with vector control. (g–h) KEGG (g) and GO (h) enrichment, highlighting translation‐related terms; color indicates rich factor and bars denote −log10(FDR). (i) Puromycin incorporation assays showing nascent protein synthesis in A549 cells with THUMPD1 overexpression (left) and knockdown (right). Values are mean ± s.d. of n = 3 biological replicates in a–d and i, and n = 9 biological replicates in e. Two‐tailed Student's t‐tests were used for comparisons between two groups, and one‐way ANOVA was used for comparisons among multiple groups, as appropriate. Significance: *p < 0.05; **p < 0.01; ***p < 0.001. Original unprocessed blots are shown in Figure S11.

While THUMPD1 has been identified as a co‐factor with NAT10 for ac4C modification on tRNA [8], its independent role in mediating ac4C on mRNA remains elusive. Accordingly, we isolated total mRNA and performed ac4C dot blot to assess global ac4C levels under THUMPD1 perturbation. Unexpectedly, global ac4C levels were positively coupled to THUMPD1 expression (Figure 3a–d). In line with the established methodology [24], we further quantified ac4C levels by LC‐MS/MS, which confirmed the dot blot findings (Figure 3e).

Given our findings that THUMPD1 mediates mRNA ac4C modification, alongside its established role in tRNA ac4C modification and translation [8], we reasoned that THUMPD1 might promote mRNA translation. To determine whether THUMPD1 overexpression globally enhances translation, we first performed polysome profiling. The results indicated that THUMPD1 significantly promotes global translation efficiency (Figure 3f). To further validate this finding, we conducted mRNA‐seq of control (NC) and THUMPD1‐knockdown (sh) cells. Enrichment analyses (KEGG, GO, and GSEA) consistently showed significant suppression of translation‐related pathways and functions (Figure 3g,h; Figure S3a). Accordingly, translation‐associated genes were markedly downregulated upon THUMPD1 knockdown (Figure S3b). Finally, we employed a puromycin incorporation assay to directly measure translation efficiency. Consistent with the previous results, THUMPD1 overexpression substantially increased puromycin incorporation, while knockdown decreased it (Figure 3i; Figure S3c).

Interestingly, here we found that “Steroid biosynthesis” also appeared among the enriched pathways in the KEGG analysis (Figure 3g). Although LUAD is not classically considered a hormone‐driven cancer, emerging evidence suggests that lung cancer cells possess intrinsic steroidogenic capacity, which may contribute to immune evasion and treatment resistance [25, 26]. The enrichment of this pathway upon THUMPD1 knockdown hints at a potential broader role for THUMPD1 in metabolic regulation beyond translation, warranting further investigation.

### 2.4 THUMPD1 Upregulates Insulin‐Like Growth Factor‐II Receptor (IGF2R) by Enhancing its Translation Independently of ac4C Modification

**FIGURE 4:**
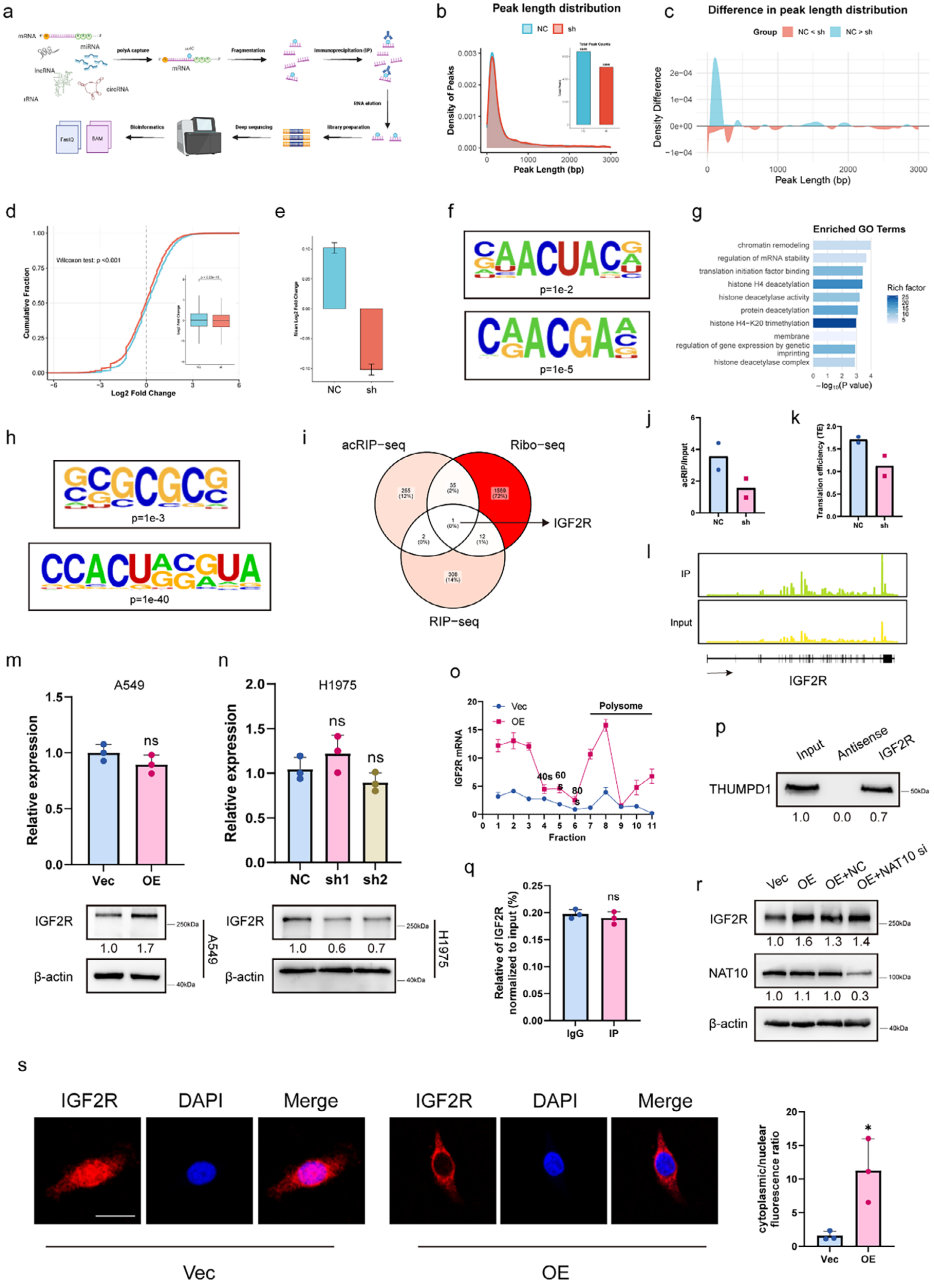
THUMPD1 upregulates IGF2R translation through an ac4C‐independent mechanism. (a) Overview of the acRIP‐seq workflow. (b–e) acRIP‐seq features and global changes upon THUMPD1 knockdown: (b) density plots of peak‐length distribution with bar charts of total ac4C peaks in NC and sh samples; (c) difference in peak‐length density (sh–NC); (d) cumulative fraction curves for NC and sh with boxplots of log2 fold change (right); (e) bar plots of mean log2 fold change in NC and sh. (f) Enriched sequence motifs identified from acRIP‐seq peaks. (g) GO enrichment of genes harboring differential ac4C peaks. (h) Sequence motifs identified from RIP‐seq peaks. (i) Integration of acRIP‐seq (reduced ac4C), RIP‐seq (THUMPD1 binding), and Ribo‐seq (reduced translation efficiency) identifies IGF2R as the sole overlapping target. (j–k) Bar plots summarizing acRIP‐seq (j) and Ribo‐seq (k) results for IGF2R. l) Genome‐browser tracks of RIP‐seq over IGF2R (IP vs Input). (m–n) qRT‐PCR and Western blot showing unchanged IGF2R mRNA but altered IGF2R protein upon THUMPD1 overexpression (m) or knockdown (n). (o) Polysome fractionation followed by qRT‐PCR showing increased association of IGF2R mRNA with heavy polysomes upon THUMPD1 overexpression. (p) RNA pull‐down confirming direct binding between THUMPD1 protein and IGF2R mRNA. (q) acRIP‐qPCR detects no ac4C signal on IGF2R mRNA. (r) NAT10 knockdown in THUMPD1‐overexpressing cells does not affect IGF2R expression. (s) RNA FISH (scale bar: 200 µm) showing the increased cytoplasmic distribution of IGF2R mRNA (red) upon THUMPD1 overexpression. Nuclei are counterstained with DAPI (blue). Values are mean of n = 2 biological replicates in j and k. Values are mean ± s.d. of n = 3 biological replicates in m–s. A Wilcoxon test was used in d. Two‐tailed Student's t‐tests were used for comparisons between two groups, and one‐way ANOVA was used for comparisons among multiple groups in m, n, q, s, as appropriate. Significance: *p < 0.05; ns, not significant. Original unprocessed blots are shown in Figure S11.

To identify potential ac4C‐modified mRNA targets under the regulation of THUMPD1, we performed acRIP‐seq on mRNAs isolated from control and THUMPD1‐knockdown cells (Figure 4a). A total of 11,543 ac4C peaks was identified, with a clear reduction in peak numbers upon THUMPD1 knockdown compared to the control (Figure 4b–e). Majority of these differential peaks were shorter than 500 bp in length (Figure 4b,c). Motif analysis revealed a significant enrichment of a cytidine (C)‐rich consensus sequence (Figure 4f). GO enrichment indicated that genes associated with the differential peaks were involved in translation initiation and mRNA stability, biological processes previously established to be influenced by ac4C modification (Figure 4g) [7, 8].

To further investigate how THUMPD1 influences translation efficiency, we conducted RIP‐seq and Ribo‐seq. The motif enriched in THUMPD1 RIP‐seq data also corresponded to the ac4C‐related CXXCXXCXX sequence (Figure 4h), and we identified 308 genes as potential direct binding targets of THUMPD1 (Figure 4i). By integrating these results with datasets from genes showing downregulated translational efficiency and reduced ac4C levels, we pinpointed IGF2R as the most prominent candidate (Figure 4i–l). We therefore hypothesized that IGF2R represents a key downstream target of THUMPD1.

We next sought to validate the regulation of IGF2R by THUMPD1. qRT‐PCR and western blot analysis showed that IGF2R expression was altered at the protein level, but not at the mRNA level (Figure 4m,n; Figure S4a,b), suggesting post‐transcriptional regulation. Given the documented role of THUMPD1 in promoting translation, we assessed the translational status of IGF2R mRNA using polysome profiling followed by qRT‐PCR. This experiment demonstrated a significant increase in IGF2R mRNA association with polysome fractions upon THUMPD1 overexpression, supporting enhanced translational efficiency (Figure 4o). An RNA pulldown assay confirmed a direct interaction between THUMPD1 and IGF2R mRNA (Figure 4p). Contrary to our initial hypothesis, however, acRIP‐qPCR failed to detect ac4C modification on IGF2R mRNA (Figure 4q). To further rule out any dependency on ac4C modification, we knocked down NAT10 in THUMPD1‐overexpressing cells and found that IGF2R protein expression remained unchanged (Figure 4r; Figure S4c). Notably, polysome profiling showed that NAT10 knockdown attenuated the global translational enhancement induced by THUMPD1 overexpression (Figure S4d). However, qRT‐PCR analysis of polysome fractions revealed that NAT10 knockdown did not abolish the enhanced association of IGF2R mRNA with polysomes in THUMPD1‐overexpressing cells (Figure S4e). These results collectively suggested that THUMPD1 regulates IGF2R expression independent of ac4C modification.

Given the critical link between mRNA trafficking and translational control [27], we hypothesized that THUMPD1 might influence the subcellular localization of IGF2R mRNA. Thus, we analyzed IGF2R mRNA distribution and results showed that THUMPD1 overexpression significantly enhanced the cytoplasmic distribution of IGF2R mRNA (Figure 4s) and THUMPD1 knockdown led to increased nuclear accumulation of IGF2R mRNA (Figure S4f). Taken together, these findings suggest that THUMPD1 regulates IGF2R not via ac4C modification, but by modulating the subcellular localization of its mRNA.

### 2.5 IGF2R Acts as a Recognized Tumor Suppressor in LUAD

Although IGF2R is well characterized as a receptor responsible for the degradation of IGF2 [28], its role as a tumor suppressor has been established in multiple cancer types over the years [29, 30, 31]. In LUAD, IGF2R has been reported to inhibit tumor growth and suppress disease progression [32, 33].

**FIGURE 5:**
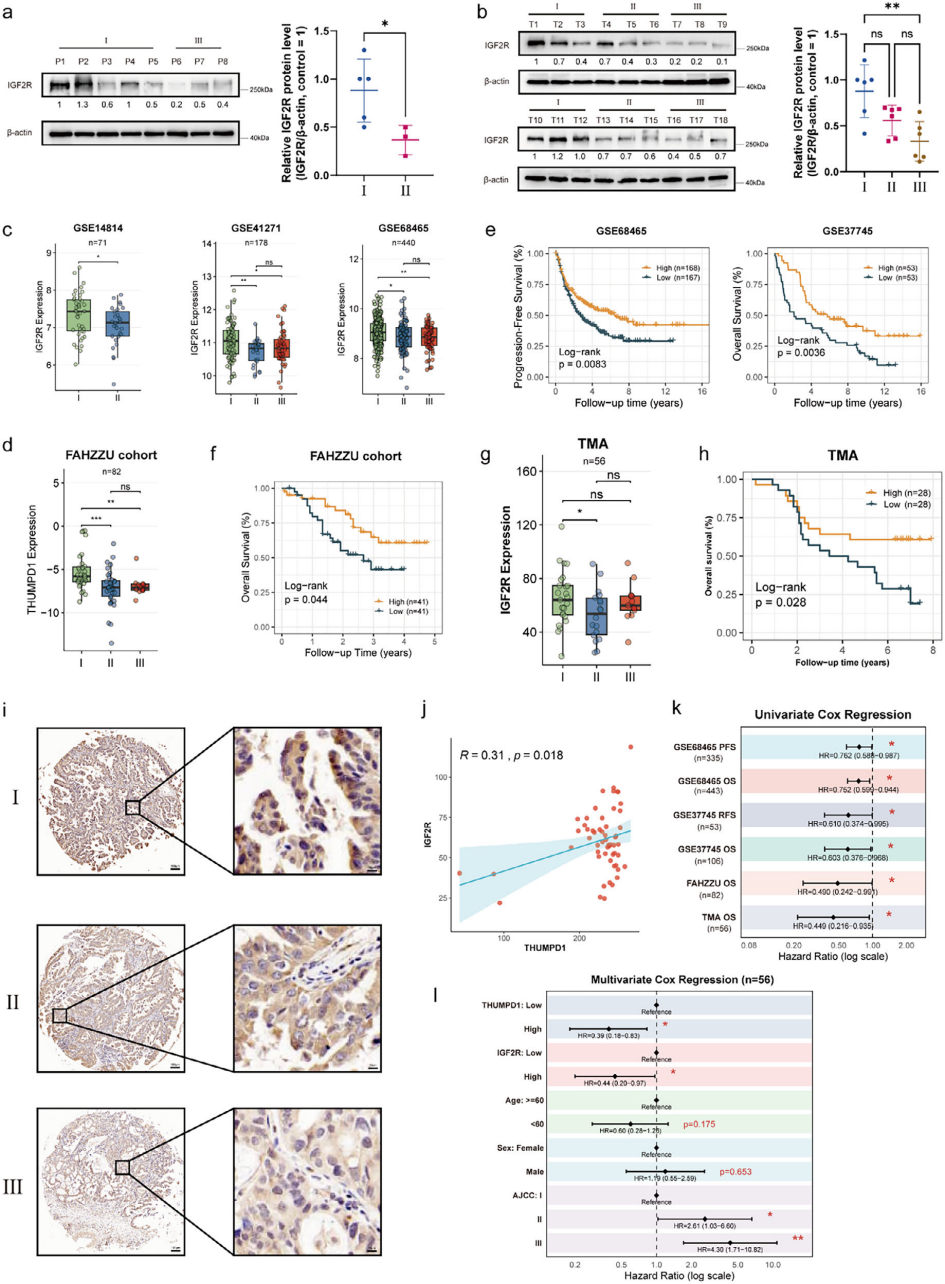
IGF2R functions as a tumor suppressor in LUAD and correlates with THUMPD1. (a) Western blot of IGF2R in LUAD tissues from AJCC stage I and III patients (n = 8) with β‐actin loading control; quantitative densitometry of IGF2R protein levels (right). (b) Western blot of IGF2R across additional primary tumors (stage I/II/III indicated, n = 18); quantitative densitometry of IGF2R protein levels (right). (c‐d) IGF2R expression by AJCC stage in external GEO cohorts (GSE14814, n = 71; GSE41271, n = 178; GSE68465, n = 440) (c) and in the FAHZZU cohort (n = 82) (d). (e‐f) Kaplan–Meier curves for progression‐free survival (PFS) and OS in GSE68465 (n = 335), GSE30219 (n = 106), and the FAHZZU cohort (n = 82), stratified by high versus low IGF2R expression. (g) TMA IGF2R expression (H‐score) by stage (I–III; n = 56). (h) TMA cohort survival by IGF2R expression (high vs low; n = 56). (i) Representative IGF2R IHC in TMA across stages I–III with high‐magnification insets. (j) Pearson correlation analysis between THUMPD1 and IGF2R protein levels. (k) Univariate Cox regression forest plot for IGF2R across independent datasets, with sample sizes indicated in the corresponding panels. (l) Multivariable Cox regression in the TMA cohort. Values are mean ± s.d. of n = 3 biological replicates in a and b. Two‐tailed Student's t‐tests were used in a. One‐way ANOVA was used in b. Wilcoxon or Kruskal–Wallis tests were used in c, d and g, as appropriate. Log‐rank tests were used in e, f and h. Univariate and multivariable Cox proportional hazards regression models were used in k and l, respectively. Significance: *p < 0.05; **p < 0.01; ***p < 0.001; ns, not significant. Original unprocessed blots are shown in Figure S11.

To validate these findings, we first performed western blot to assess IGF2R expression across different tumor stages using the eight samples previously subjected to LC‐MS/MS (Figure 1a). Interestingly, IGF2R expression was significantly downregulated in stage III tissues (Figure 5a), which was further confirmed in additional clinical samples (Figure 5b). Similar results were also observed in publicly available datasets and in the FAHZZU cohort (Figure 5c,d; Figure S5a). Moreover, higher IGF2R expression was associated with improved overall survival in some independent cohorts (Figure 5e,f; Figure S5b). Although the difference between stages II and III did not reach statistical significance in TMA cohort, IGF2R expression was significantly lower in stage II than in stage I, and reduced IGF2R levels predicted poorer prognosis (Figure 5g,h). In contrast to the predominant nuclear localization of THUMPD1, IGF2R was mainly detected in the cytoplasm and membrane (Figure 5i). A positive correlation between THUMPD1 and IGF2R protein expression was observed (Figure 5j), suggesting that the regulatory influence of THUMPD1 may depend on cytoplasmic distribution (Figure 4s). Univariate and multivariate Cox regression analysis identified IGF2R as a favorable prognostic factor (Figure 5k; Figure S5c‐e). Finally, multivariate Cox analysis in the TMA cohort demonstrated that both THUMPD1 and IGF2R were independent prognostic factors (Figure 5l). In the FAHZZU cohort, both markers retained prognostic significance after adjustment for the available non‐collinear covariates, although AJCC stage was not included in the model because of collinearity (Figure S5f).

### 2.6 IGF2R Mediates the Tumor‐Suppressive Function of THUMPD1

**FIGURE 6:**
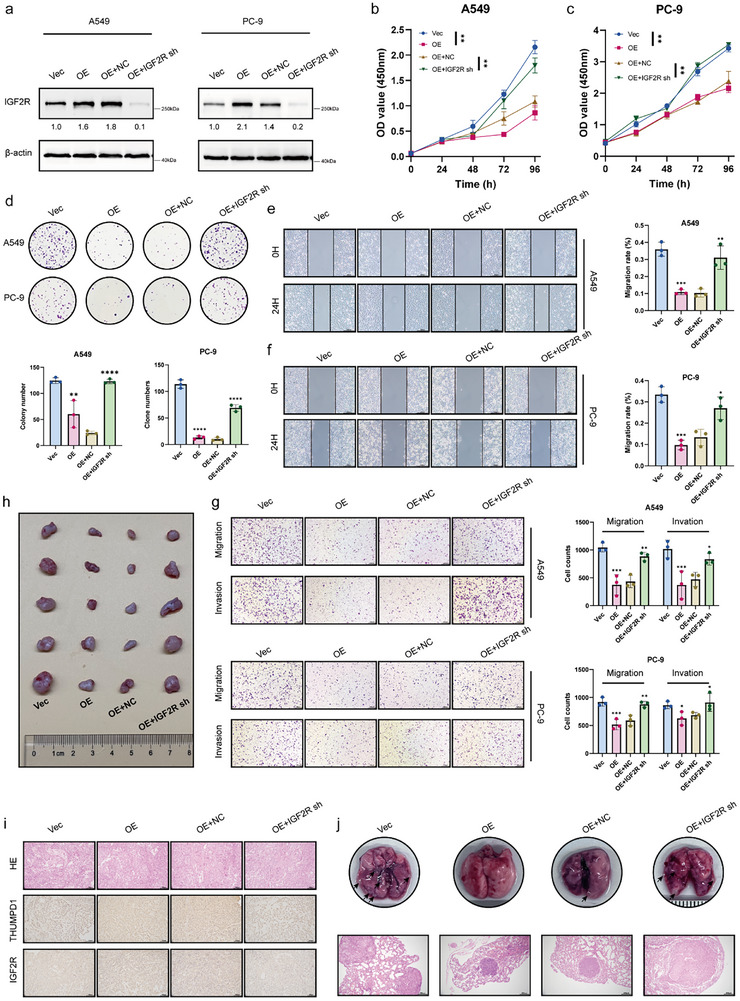
IGF2R mediates the tumor‐suppressive effects of THUMPD1. (a) Western blot confirming IGF2R knockdown in THUMPD1‐overexpressing cells. (b–c) CCK‐8 growth curves for A549 (b) and PC‐9 (c). (d) Colony‐formation assays in A549 and PC‐9 with representative images and quantification. (e–f) Wound‐healing assays (0 h, 24 h) with migration‐rate quantification in A549 (e) and PC‐9 (f). (g) Transwell migration and invasion assays with representative fields and quantification in A549 and PC‐9. (h) Image of subcutaneous xenograft tumors. (i) Representative haematoxylin and eosin (HE) staining and IHC for THUMPD1 and IGF2R in xenograft tumors. (j) Experimental lung‐metastasis models showing representative gross lung images, with black arrows indicating pulmonary metastatic nodules and corresponding HE sections. Values are mean ± s.d. of n = 3 biological replicates in a–g. Xenograft and metastasis experiments were performed with n = 5 mice per group in h–j. One‐way ANOVA was used for comparisons among multiple groups. Significance: *p < 0.05; **p < 0.01; ***p < 0.001; ****p < 0.0001. Original unprocessed blots are shown in Figure S11.

To validate the functional significance of IGF2R as a key downstream effector of THUMPD1 in LUAD, we performed rescue experiments by knocking down IGF2R in THUMPD1‐overexpressing cells (Figure 6a). Results showed that knockdown of IGF2R significantly attenuated the suppressive effects of THUMPD1 overexpression on tumor cell growth and viability (Figure 6b–d). Similarly, the inhibition of cell migration and invasion by THUMPD1 was also partially reversed upon IGF2R knocked down (Figure 6e–g). We further extended these findings in vivo. Consistent with the in vitro data, IGF2R knockdown partially restored tumor growth and volume and led to an increase in lung metastatic nodules in mouse models (Figure 6h–j and Figure S6a–d). Taken together, these rescue experiments demonstrated that the tumor‐suppressive function of THUMPD1 is mediated through the positive regulation of IGF2R expression.

### 2.7 Mitophagy Serves as a Critical Mechanism of THUMPD1‐IGF2R‐Mediated Tumor Suppression

**FIGURE 7:**
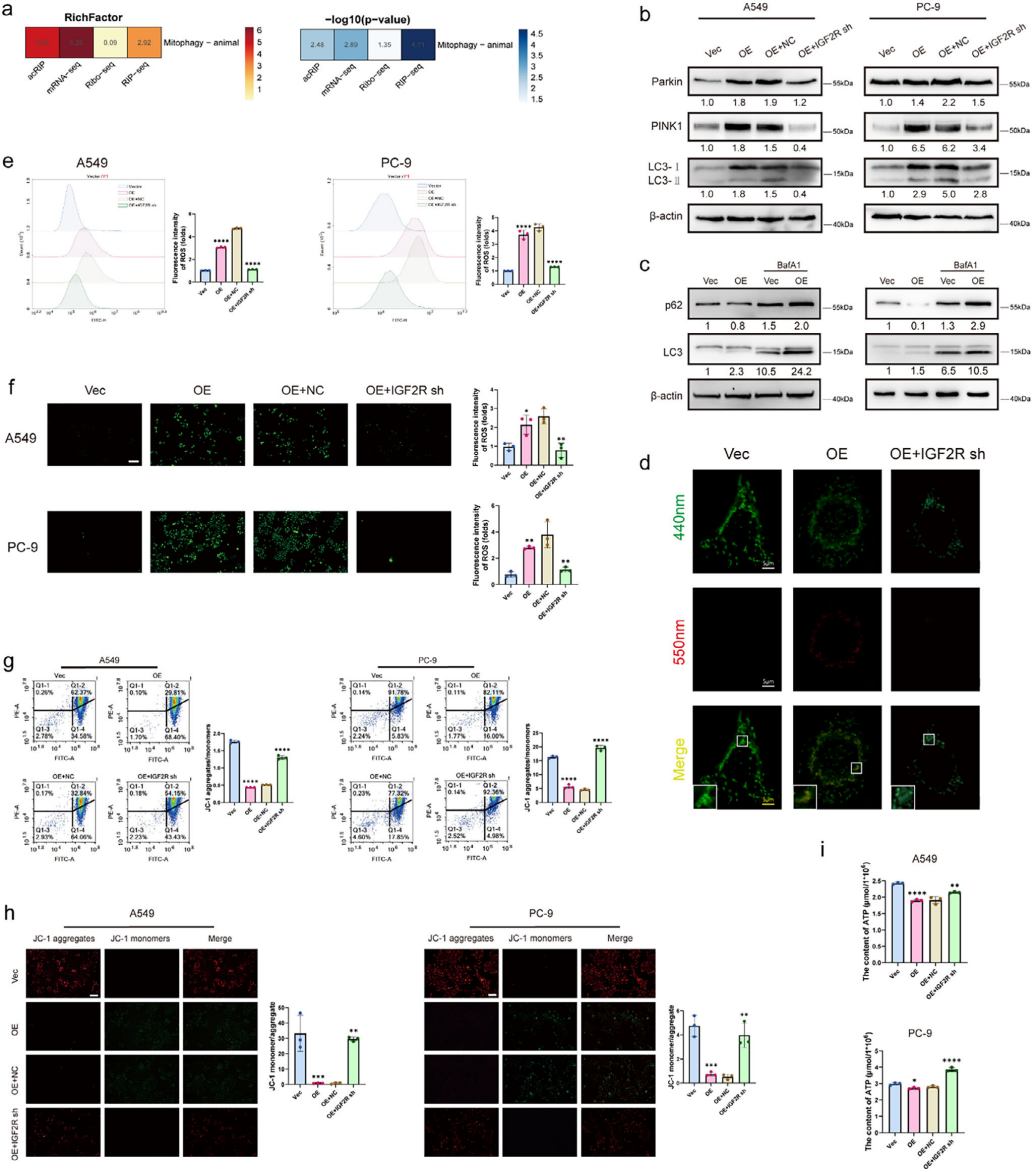
THUMPD1–IGF2R induces mitophagy‐related stress. (a) KEGG pathway mitophagy enrichment analysis across multi‐omics sequencing datasets. Left: enrichment scores. Right: ‐log10(p‐value). (b) Western blot for PINK1, Parkin, and LC3 (LC3‐I/II). (c) Western blot for p62 and LC3 in A549 and PC‐9 cells under control and THUMPD1‐overexpression conditions, with or without bafilomycin A1 (BafA1). (d) Representative mt‐Keima fluorescence images in the indicated groups. (e) Flow‐cytometry histograms of intracellular ROS with quantification. (f) Representative fluorescence images (scale bar: 200 µm) of DCF staining for ROS with corresponding intensity quantification. (g) JC‐1 flow‐cytometry analysis of mitochondrial membrane potential with quantification. (h) JC‐1 microscopy (scale bar: 200 µm) showing aggregates (red)/monomers (green) and quantification. (i) ATP content in A549 and PC‐9. Values are mean ± s.d. of n = 3 biological replicates in b‐i. One‐way ANOVA was used for comparisons among multiple groups. Significance: *p < 0.05; **p < 0.01; ***p < 0.001; ****p < 0.0001. Original unprocessed blots for panel b are shown in Figure S11, whereas those for panel c are shown in Figure S12.

Having established the THUMPD1–IGF2R axis, we next sought to identify the downstream process responsible for this effect. KEGG analysis indicated a consistent enrichment of mitophagy‐related terms across all four sequencing datasets (Figure 7a), prompting a detailed investigation into this pathway.

Mitophagy is a selective autophagic process that targets damaged mitochondria for lysosomal degradation when mitochondrial damage surpasses repair capacity, thereby supporting mitochondrial homeostasis and cell survival [34]. Recent studies have highlighted its diverse roles in various cancers [35, 36, 37]. A key regulatory mechanism involves the PTEN‐induced kinase 1 (PINK1)–parkin RBR E3 ubiquitin‐protein ligase (Parkin) axis [34]. Additionally, microtubule‐associated protein 1 light chain 3 (LC3) lipidation marks autophagosome formation and expansion, facilitating the engulfment of ubiquitin‐tagged mitochondria and subsequent lysosomal degradation [34]. We therefore examined these mitophagy markers in LUAD cell lines. Consistent with the pathway analysis, their expression was positively correlated with THUMPD1 (Figure 7b; Figure S7a).

To further assess mitophagic flux, we treated cells with the lysosomal inhibitor bafilomycin A1 (BafA1). Under these conditions, LC3‐II and p62 accumulated to a greater extent in THUMPD1‐overexpressing cells, supporting enhanced mitophagic flux (Figure 7c). To visualize mitophagy induction more directly, we employed the quantitative mitophagy reporter mt‐Keima. Consistent with the BafA1 results, mt‐Keima imaging showed that THUMPD1 overexpression substantially increased the number of red puncta per cell, indicating enhanced delivery of mitochondria to the acidic lysosomal compartment during mitophagy, whereas IGF2R knockdown markedly reduced this effect (Figure 7d).

Because mitophagy is typically triggered by mitochondrial damage, we next examined mitochondrial stress‐related phenotypes. THUMPD1 overexpression elevated reactive oxygen species (ROS) (Figure 7e,f) and reduced mitochondrial membrane potential (Figure 7g,h), as assessed by JC‐1 staining, in which a decreased red/green fluorescence ratio indicates mitochondrial depolarization. Both effects were rescued by IGF2R knockdown.

To test whether THUMPD1 knockdown confers resistance to mitophagy induction, we treated cells with simvastatin, the mitophagy inducer. Under these conditions, THUMPD1 knockdown attenuated the simvastatin‐induced ROS increase and mitochondrial membrane potential loss (Figure S7b–e). Given the central role of mitochondria in ATP production, we also measured ATP levels. THUMPD1 knockdown increased ATP production, while THUMPD1 overexpression decreased it, an effect reversed by IGF2R knockdown (Figure 7i; Figure S7f).

Since mitophagy is typically associated with promoting tumor progression by maintaining mitochondrial health in cancer cells [37], the concurrent enhancement of mitophagy and suppression of tumor growth following THUMPD1 overexpression was unexpected. This finding led us to hypothesize that THUMPD1 activates a distinct, tumor‐suppressive form of mitophagy, and we aimed to uncover its underlying mechanism.

### 2.8 Cu^+^ Overload and Cuproptosis‐Associated Metabolic Stress Activate Excessive Mitophagy in LUAD

**FIGURE 8:**
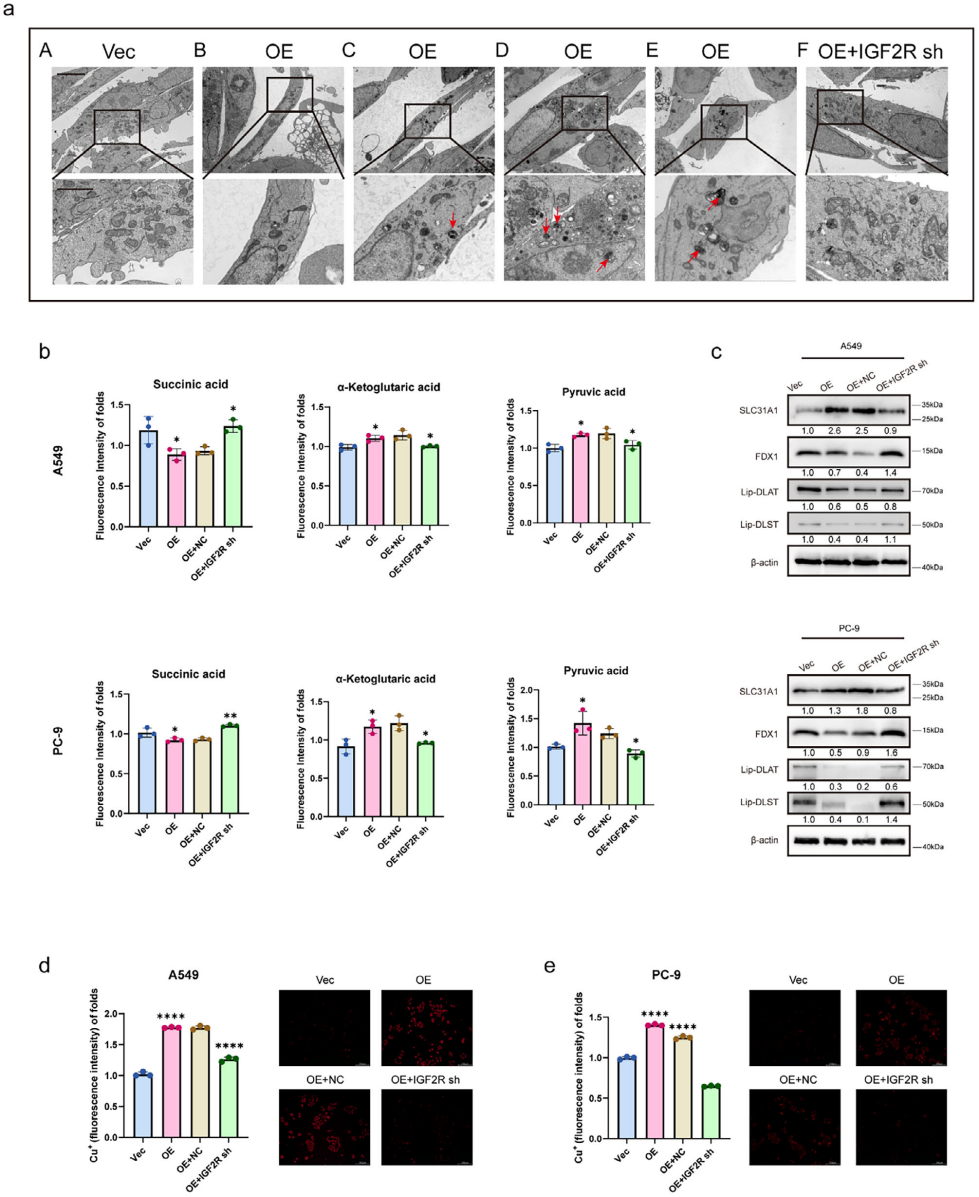
Cu^+^ overload and cuproptosis‐associated metabolic stress promote excessive mitophagy in LUAD. (a) Transmission electron microscopy (TEM) images. Top row: overview (scale bar: 50 µm); bottom row: magnified views (scale bar: 20 µm) showing progressive mitochondrial damage and mitophagosome formation (red arrows) in THUMPD1‐overexpressing (OE) cells compared to vector control (Vec), with partial rescue upon IGF2R knockdown (OE + IGF2R sh). Images B–E (OE group) are all from the same OE sample, representing different stages of mitochondrial damage. (b) Validation of central‐carbon metabolites changes in A549 and PC9 cells by assay kits. (c) Western blot of SLC31A1, FDX1, and lipoylated DLAT/DLST. (d‐e) Intracellular Cu^+^ measurements in A549 (d) and PC‐9 (e) cells across the indicated groups. Left: quantitative Cu^+^ levels measured by assay kit. Right: representative CopperSensor‐1 (CS1) fluorescence images. Values are mean ± s.d. of n = 3 biological replicates in b‐e. One‐way ANOVA was used for comparisons among multiple groups. Significance: *p < 0.05; **p < 0.01; ****p < 0.0001. Original unprocessed blots are shown in Figure S12.

To visually evaluate the ultrastructural alterations resulting from THUMPD1 overexpression, transmission electron microscope was performed. This analysis uncovered a sequence of pronounced morphological changes. In vector control cells, mitochondria exhibited a typical healthy morphology (Figure 8a, A). In contrast, THUMPD1‐overexpressing cells displayed a marked pathological progression (Figure 8a, B–E). Specifically, we observed varying degrees of mitochondrial impairment, beginning with a reduced number of mitochondria and loss of cristae structure (Figure 8a, B), followed by the appearance of sporadic double‐membraned autophagic vesicles engulfing damaged mitochondria (Figure 8a, C), which further progressed to an abundance of such vesicles (Figure 8a, D). Ultimately, the cells exhibited a rounded morphology, a hallmark of impending cell death (Figure 8a, E). Notably, knockdown of IGF2R partially ameliorated the mitophagy‐related damage (Figure 8a, F). These results illustrated a graded process in which overwhelming and dysregulated activation of mitophagy is associated with increasing cellular distress, ultimately suppressing tumor cell growth.

Given that unchecked mitophagy disrupts energy metabolism, we profiled the resulting metabolic changes. Succinic acid, α‐ketoglutaric acid, and pyruvic acid exhibited opposing trends in the Vec vs OE and OE vs OE+IGF2R sh comparisons (Figure S8a). These findings were further validated using commercial assay kits (Figure 8b; Figure S8b). A recently identified copper‐dependent cell death mechanism, cuproptosis, involves Cu^+^‐mediated disruption of lipoylated tricarboxylic acid cycle (TCA) proteins [38]. This process is initiated when Cu^+^ directly binds to lipoylated components of the pyruvate dehydrogenase (PDH) and α‐ketoglutarate dehydrogenase (KDH) complexes, inhibiting their activity. The resulting blockade prevents the conversion of pyruvate to acetyl‐CoA and α‐ketoglutarate to succinyl‐CoA, leading to accumulation of pyruvate and α‐ketoglutarate, depletion of succinate, and ultimately metabolic collapse (Figure S8c), a profile consistent with our observations. We therefore hypothesized that Cu^+^ overload induces intense mitophagy and consequent cell death.

Among cuproptosis‐related genes, only solute carrier family 31 member 1 (SLC31A1) was downregulated upon THUMPD1 knockdown in our mRNA‐seq data. Western blot confirmed that SLC31A1 was elevated in THUMPD1‐overexpressing cells and restored by IGF2R knockdown (Figure 8c), while being reduced in THUMPD1‐knockdown cells (Figure S8d). We also examined ferredoxin 1 (FDX1), an upstream regulator of protein lipoylation, along with lipoylation levels of dihydrolipoamide S‐acetyltransferase (DLAT) and dihydrolipoamide S‐succinyltransferase (DLST). Expression of these key enzymes correlated with FDX1, indicating disruption of normal TCA cycle function (Figure 8c; Figure S8d). Since SLC31A1 is a copper importer, we measured Cu^+^ levels and observed an increase upon THUMPD1 overexpression and a decrease upon its knockdown (Figure 8d; Figure S8e). To visualize intracellular copper accumulation, we further stained cells with the fluorescent Cu^+^ probe CopperSensor‐1 (CS1), which revealed stronger copper‐associated fluorescence in THUMPD1‐overexpressing cells and reduced signal upon IGF2R knockdown, consistent with the quantitative Cu^+^ measurement (Figure 8d,e; Figure S8f).

Together, these data support a model in which THUMPD1 promotes Cu^+^ accumulation via SLC31A1, triggering excessive mitophagy and metabolic disruption in LUAD cells.

### 2.9 Elesclomol (ES) Inhibits LUAD by Inducing Cuproptosis

**FIGURE 9:**
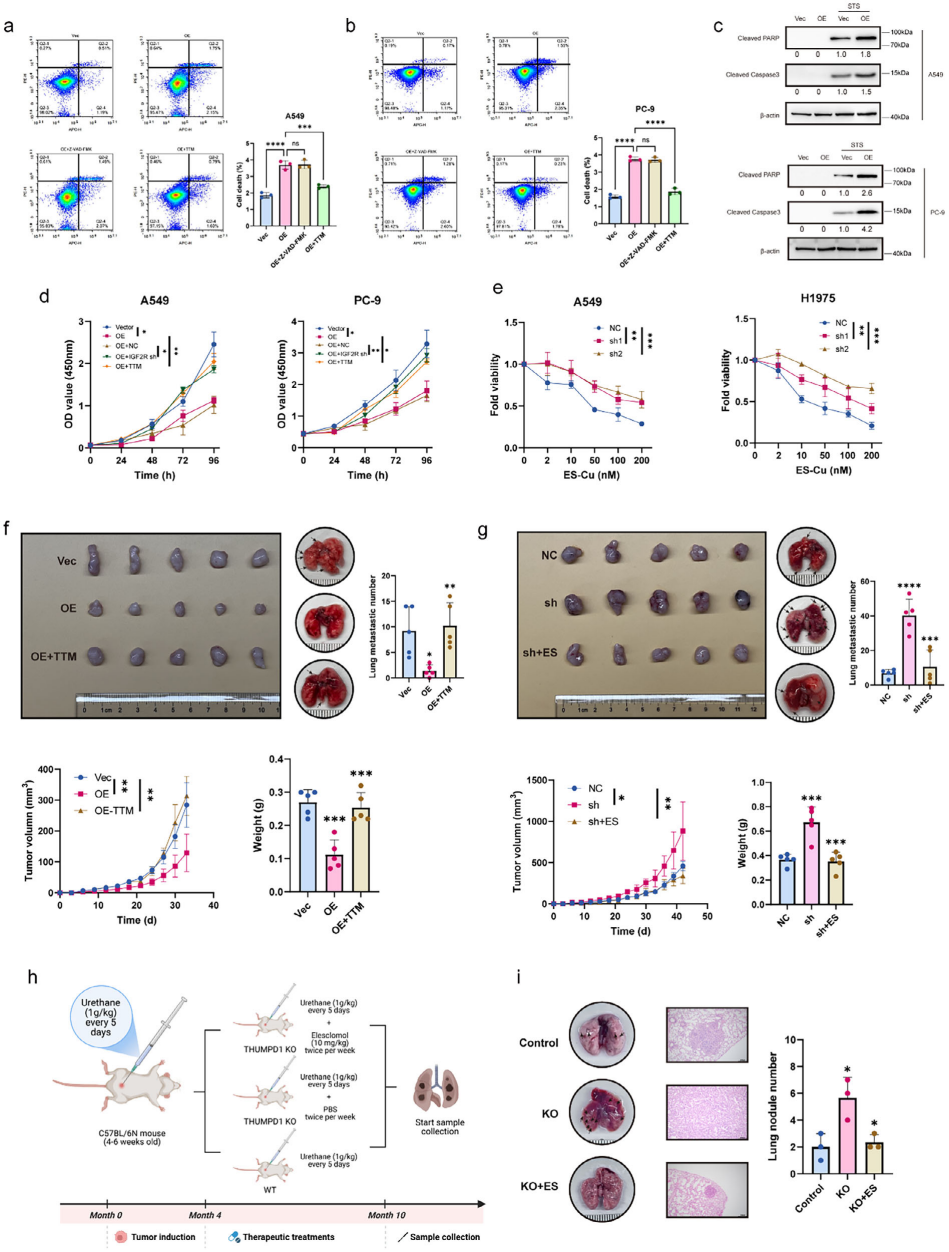
THUMPD1‐mediated copper‐dependent cell death is functionally validated in vitro and in vivo. (a–b) Annexin V/PI flow‐cytometry analysis of cell death in A549 (a) and PC‐9 (b) cells showing the effects of THUMPD1 overexpression and its modulation by the pan‐caspase inhibitor Z‐VAD‐FMK and the copper chelator tetrathiomolybdate (TTM), with representative plots and corresponding quantification. (c) Western blot of cleaved PARP and caspase‐3 in A549 and PC‐9 cells under control and THUMPD1‐overexpression conditions, with or without staurosporine (STS). (d) CCK‐8 assays in A549 and PC‐9 cells showing the effects of THUMPD1 overexpression, IGF2R knockdown or TTM treatment in the THUMPD1‐overexpression background. (e) CCK‐8 assays in A549 and H1975 cells with THUMPD1 knockdown treated with the indicated concentrations of elesclomol (ES)–Cu (1:1). (f) In vivo TTM treatment in the THUMPD1‐overexpression setting, including representative image of subcutaneous xenograft tumors, tumor‐growth curves, tumor weight, representative lung images from tail‐vein metastasis assays, black arrows indicating pulmonary metastatic nodules, and quantification of lung metastatic burden in the indicated groups. (g) In vivo ES treatment in the THUMPD1‐knockdown setting, including representative image of subcutaneous xenograft tumors, tumor‐growth curves, tumor weight, representative lung images from tail‐vein metastasis assays, black arrows indicating pulmonary metastatic nodules, and quantification of lung metastatic burden in the indicated groups. (h) Schematic of the *Thumpd1*‐knockout (KO) urethane‐induced LUAD model and treatment schedule with ES. (i) Representative lung images, black arrows indicating pulmonary metastatic nodules, HE staining of lung tumor lesions, and quantification of pulmonary tumor nodules in the KO mouse model among the indicated groups. Values are mean ± s.d. of n = 3 biological replicates in a–e. Xenograft and metastasis experiments were performed with n = 5 mice per group in f and g, and the KO mouse experiment was performed with n = 3 mice per group in i. One‐way ANOVA was used for comparisons among multiple groups. Significance: *p < 0.05; **p < 0.01; ***p < 0.001; ****p < 0.0001; ns, not significant. Original unprocessed blots are shown in Figure S12.

To further determine whether THUMPD1‐induced growth suppression is accompanied by increased cell death, we quantified cell death by Annexin V/PI flow cytometry. THUMPD1 overexpression significantly increased the fraction of dead cells. Notably, this increase was not reversed by the pan‐caspase inhibitor Z‐VAD‐FMK, but was markedly attenuated by the copper chelator tetrathiomolybdate (TTM), a copper chelator that limits bioavailable copper and has been widely used to suppress copper‐dependent cell death (cuproptosis), [38, 39] suggesting that THUMPD1‐induced cell death is not primarily mediated by classical caspase‐dependent apoptosis but is instead associated with a copper‐dependent death program (Figure 9a,b). In parallel, western blot showed that cleaved caspase‐3 and cleaved PARP were not detectable under basal conditions, whereas staurosporine (STS) induced robust cleavage as a positive control (Figure 9c). These findings suggest that THUMPD1‐induced cell death is not dominated by classical caspase‐dependent apoptosis and is instead consistent with a copper‐dependent death program.

We next examined whether altering copper availability could modulate the growth phenotype driven by THUMPD1. TTM attenuated the THUMPD1‐overexpression‐induced growth suppression and partially restored cell viability (Figure 9d). Conversely, when THUMPD1‐knockdown cells were exposed to ES‐Cu (1:1), they displayed relative resistance to the treatment compared with control cells (Figure 9e). Together, these findings further support that THUMPD1‐mediated inhibition of LUAD is closely associated with a copper‐dependent death program consistent with cuproptosis.

In line with in vitro findings, xenograft and metastasis rescue experiments further supported the functional significance of copper‐dependent tumor suppression. Compared with the control, THUMPD1 overexpression markedly reduced subcutaneous tumor growth and pulmonary metastatic burden, whereas TTM treatment partially reversed these inhibitory effects, as reflected by increased tumor volume, tumor weight, and lung metastatic nodules (Figure 9f). Importantly, measurements of intratumoral copper levels showed that TTM treatment reduced copper accumulation in tumors (Figure S9a). Western blot of tumor tissues further showed that FDX1, DLAT, and DLST were decreased upon THUMPD1 overexpression and altered after TTM treatment, consistent with the corresponding in vitro findings (Figure S9b).

Conversely, compared with the control, THUMPD1 knockdown significantly promoted subcutaneous tumor growth and lung metastatic colonization, whereas treatment with ES partially suppressed these tumor‐promoting effects (Figure 9g). Consistent with its role as a copper ionophore, ES increased intratumoral copper levels (Figure S9c). In parallel, FDX1, DLAT, and DLST were elevated upon THUMPD1 knockdown and altered after ES treatment (Figure S9d). These results further support that THUMPD1‐mediated tumor suppression is, at least in part, dependent on copper availability and can be pharmacologically modulated by agents that alter copper homeostasis.

Finally, we evaluated the antitumor activity of ES in a genetically engineered and chemically induced in vivo model. Whole‐body *Thumpd1*‐knockout (KO) mice were generated (Figure S9e), and LUAD was induced with urethane for 10 months. Beginning at month 4, KO mice were divided into two groups and received intraperitoneal injection of ES (10 mg/kg) and PBS respectively (Figure 9h). At the endpoint, KO mice developed more lung nodules than controls, whereas ES reduced lung tumor burden compared with KO vehicle controls, demonstrating in vivo efficacy (Figure 9i). Together, these findings support a critical role for THUMPD1 in regulating copper‐dependent tumor suppression in LUAD and suggest that ES may represent a potential therapeutic strategy for THUMPD1‐low tumors.

### 2.10 THUMPD1 Mediates Cuproptosis and Mitophagy via IGF2R‐Dependent Inhibition of Protein kinase B (AKT) and Subsequent Activation of AMP‐Activated Protein kinase (AMPK) Signaling

**FIGURE 10:**
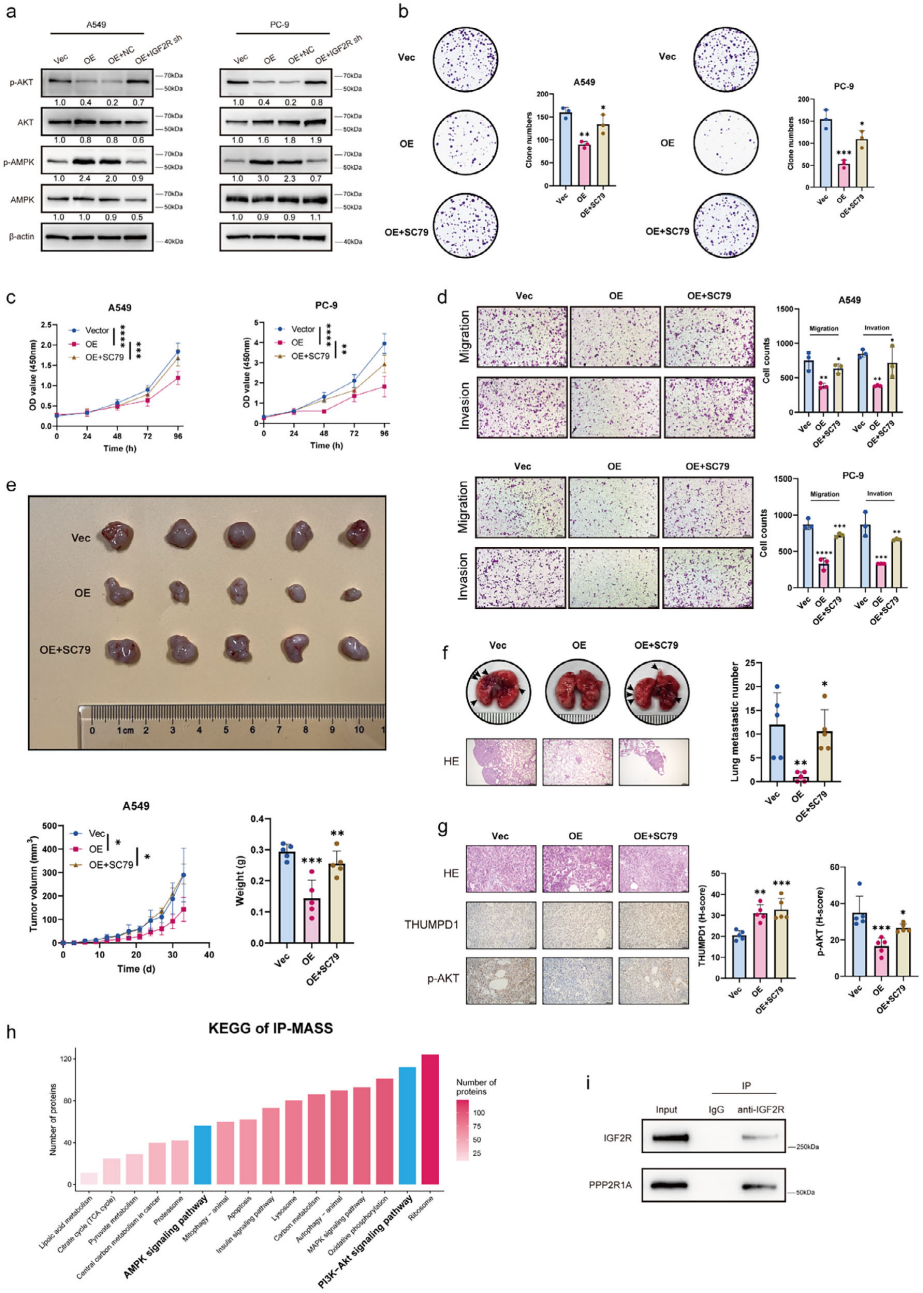
THUMPD1 mediates cuproptosis and mitophagy via IGF2R‐dependent inhibition of AKT and activation of AMPK. (a) Western blot of AKT/AMPK pathway proteins in A549 and PC‐9 cells under the indicated conditions. (b) Colony‐formation rescue assays in A549 and PC‐9 cells treated with the AKT activator SC79, with representative images and quantification. (c) CCK‐8 rescue assays in A549 and PC‐9 cells treated with SC79. (d) Transwell migration and invasion assays with SC79 treatment in A549 and PC‐9 cells, with representative fields and quantification. (e) In vivo rescue experiments with SC79 treatment, including image of subcutaneous xenograft tumors, tumor‐growth curves and tumor weight quantification in the indicated groups. (f) Representative lung images, with black arrows indicating pulmonary metastatic nodules, HE staining, and quantification of pulmonary metastatic nodules in the indicated groups. (g) Representative HE staining and IHC for THUMPD1 and p‐AKT in xenograft tumors, with corresponding H‐score quantification. (h) KEGG enrichment analysis of IGF2R‐associated proteins identified by IP‐MS. (i) Co‐immunoprecipitation followed by Western blot confirming the interaction between IGF2R and PPP2R1A. Values are mean ± s.d. of n = 3 biological replicates in a–d and i. Xenograft and metastasis experiments were performed with n = 5 mice per group in e–g. One‐way ANOVA was used for comparisons among multiple groups. Significance: *p < 0.05; **p < 0.01; ***p < 0.001; ****p < 0.0001. Original unprocessed blots are shown in Figure S12.

We previously showed that THUMPD1 upregulates IGF2R by relocalizing IGF2R mRNA. How IGF2R connects to cuproptosis and mitophagy, however, remained unclear. In this axis, SLC31A1 is pivotal because Cu^+^ influx perturbs downstream metabolism. Prior studies indicate that AMPK stabilizes SLC31A1 [40], and IGF2R can engage AKT signaling [30, 41]. Based on this, we hypothesized that the THUMPD1/IGF2R axis inhibits LUAD via inhibition of AKT and activation of AMPK signaling that sustains SLC31A1 and couples cuproptosis to mitophagy. Western blot showed that THUMPD1 overexpression reduced AKT phosphorylation and enhanced AMPK activation, whereas IGF2R knockdown reversed these effects (Figure 10a; Figure S10a).

AKT reactivation was next used to functionally validate the role of this pathway in THUMPD1‐mediated tumor suppression. Western blot analysis confirmed that SC79 treatment effectively reactivated AKT signaling (Figure S10b). Treatment of THUMPD1‐overexpressing cells with the AKT activator SC79 markedly attenuated the suppressive effects of THUMPD1 overexpression on colony formation, cell viability, migration, and invasion (Figure 10b–d). In vivo, SC79 likewise partially rescued the inhibitory effects of THUMPD1 overexpression on subcutaneous tumor growth and lung metastatic nodules (Figure 10e,f). Consistently, immunohistochemistry of xenograft tissues confirmed reduced p‐AKT levels in THUMPD1‐overexpressing tumors (Figure 10g). Together, these results indicate that AKT inhibition is functionally required for THUMPD1‐mediated tumor suppression.

To gain mechanistic insight into how IGF2R suppresses AKT signaling, we performed IGF2R co‐immunoprecipitation coupled with mass spectrometry. KEGG enrichment analysis of the identified proteins revealed significant enrichment of pathways highly consistent with our mechanistic model, including phosphoinositide 3‐kinase (PI3K)‐Akt signaling, AMPK signaling, autophagy, mitophagy, lysosome, proteasome, oxidative phosphorylation, citrate cycle, and pyruvate metabolism (Figure 10h). Among the candidate interactors, protein phosphatase 2 scaffold subunit A alpha (PPP2R1A) was of particular interest because it encodes the Aα scaffold subunit of protein phosphatase 2A (PP2A), a major serine/threonine phosphatase known to restrain AKT pathway activity [42]. Previous studies have shown that disruption of PPP2R1A/PP2A function results in hyperphosphorylation of AKT and related oncogenic signaling nodes, supporting a role for PPP2R1A‐containing PP2A complexes in AKT dephosphorylation [43, 44]. Co‐immunoprecipitation followed by western blot further confirmed the interaction between IGF2R and PPP2R1A (Figure 10i). These findings suggest that IGF2R may inhibit AKT signaling, at least in part, through recruitment or stabilization of PPP2R1A/PP2A‐associated phosphatase activity.

**FIGURE 11:**
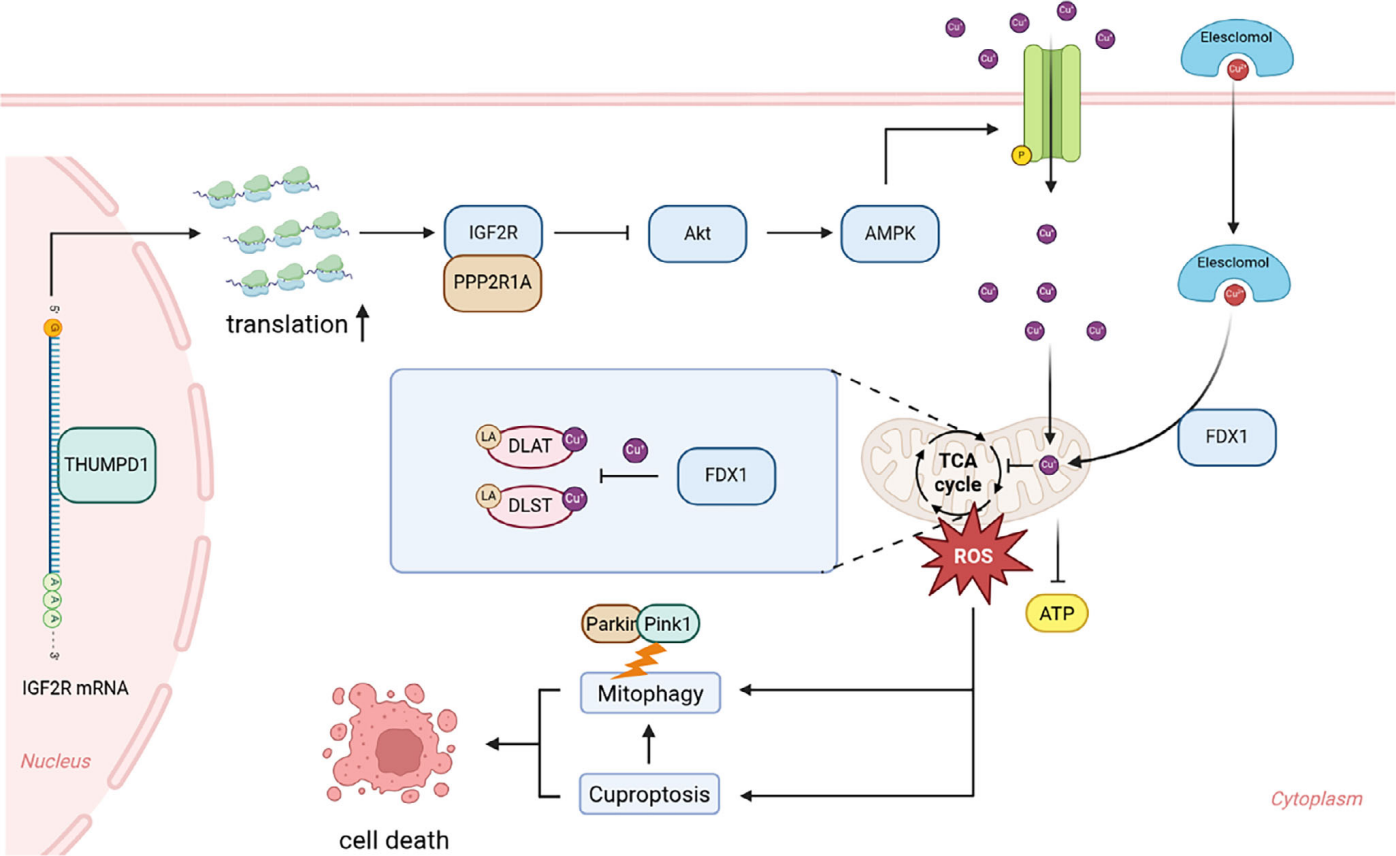
Proposed schematic model of the tumor‐suppressive mechanism mediated by the THUMPD1/IGF2R signaling axis in LUAD. THUMPD1 binds to IGF2R mRNA in the nucleus and promotes its export to the cytoplasm, thereby enhancing IGF2R translation. The increased IGF2R protein suppresses AKT signaling, at least in part through interaction with PPP2R1A, which may facilitate PP2A‐associated dephosphorylation of AKT, leading to activation of AMPK. Activated AMPK stabilizes and upregulates the copper transporter SLC31A1, resulting in elevated intracellular Cu^+^ accumulation. Excess Cu^+^ induces cuproptosis by disrupting lipoylated TCA cycle proteins and mitochondrial metabolism, while simultaneously triggering excessive mitophagy. The combined effects of copper‐dependent cell death and uncontrolled mitophagy ultimately suppress LUAD cell proliferation and metastasis. Created with BioRender.com.

In sum, our data delineate a signaling cascade in which the THUMPD1–IGF2R axis inhibits AKT and activates AMPK pathway to upregulate SLC31A1, thereby inducing cuproptosis and mitophagy, which collectively constrain LUAD (Figure 11).

## 3 Discussion

In this study, we have systematically delineated a novel molecular pathway through which the THUMPD1–IGF2R signaling axis suppresses LUAD progression by inhibiting AKT signaling, which in turn activates AMPK and upregulates SLC31A1, thereby inducing cuproptosis and excessive mitophagy. It is also noteworthy that we identified, for the first time, a role for THUMPD1 in mediating ac4C modifications on mRNA, a finding that warrants further validation in future studies.

Current understanding of THUMPD1 in cancer remains limited, and prior studies have reported inconsistent conclusions regarding whether THUMPD1 is required for NAT10‐mediated ac4C deposition on mRNA [7, 14]. Given the generally low abundance of ac4C in mRNA and the methodological challenges associated with its detection, it is important to interpret mRNA ac4C regulation using orthogonal approaches. In our study, acRIP‐seq performed on poly(A)+‐enriched RNA revealed a broad reduction of ac4C peaks upon THUMPD1 knockdown, supporting a role for THUMPD1 in shaping the ac4C landscape of mRNA. Consistently, global ac4C signals detected by dot blot and LC–MS/MS also changed with THUMPD1 perturbation. However, because the RNA used for dot blot and LC–MS/MS analyses was obtained using a kit that enriches, but does not strictly purify, mRNA, these global measurements should be interpreted with caution. At the same time, our mechanistic data reveal an ac4C‐independent function of THUMPD1, because IGF2R mRNA did not show detectable ac4C enrichment by acRIP‐qPCR and NAT10 depletion did not abolish THUMPD1‐mediated upregulation of IGF2R protein. These findings suggest that THUMPD1 can regulate gene expression through both ac4C‐associated and ac4C‐independent mechanisms, and that the latter is critical for the THUMPD1–IGF2R axis in LUAD.

Consistent with this ac4C‐independent mechanism, THUMPD1 increased IGF2R protein abundance without altering IGF2R mRNA levels, indicating translational regulation. Mechanistically, THUMPD1 directly binds IGF2R mRNA and facilitates its nuclear export, thereby increasing the cytoplasmic pool of translatable IGF2R transcripts and enhancing IGF2R translation. This mode of regulation reveals a previously unrecognized function for THUMPD1 in controlling mRNA localization and translational efficiency, expanding its functional repertoire beyond its established role in tRNA modification. The identification of IGF2R as a direct translational target of THUMPD1 provides a mechanistic basis for its tumor‐suppressive effects in LUAD and establishes a novel paradigm for THUMPD1‐mediated gene regulation.

The role of mitophagy in cancer has been extensively studied. In general, mitophagy is a selective autophagic process through which cells encapsulate and degrade damaged mitochondria, thereby preserving mitochondrial quality and cellular homeostasis [34]. However, mitophagy exerts context‐dependent effects in cancer, capable of either suppressing or promoting tumor progression [45]. In this study, LUAD inhibition was associated with excessive mitophagic activity, prompting us to explore its link to tumor suppression.

Recent work has shown that copper exposure can induce PINK1/Parkin‐dependent mitophagy [46]. Moreover, excessive copper leads to a sharp rise in ROS levels and autophagy activation, long recognized as a major contributor to cell death [47]. Building on these findings, we observed metabolic alterations and upregulated SLC31A1 expression in our system, pointing to cuproptosis, newly defined copper‐dependent regulated cell death pathway. Crucially, we demonstrated that the ensuing mitophagy does not represent a pro‐survival adaptive response but rather constitutes an excessive and irreversible “lethal mitophagy” that drives tumor cell death. This finding elegantly explains why enhanced mitophagy in this context exerts a tumor‐suppressive effect, contrary to its conventional pro‐tumor role.

In addition, the findings of our study hold significant potential for clinical translation. We confirmed that pharmaceuticals targeting this pathway are highly effective. The cuproptosis inhibitor TTM reversed the tumor‐suppressive effects of THUMPD1 overexpression. Conversely, the cuproptosis inducer elesclomol exhibited potent tumor‐killing efficacy in vivo. This suggests a promising therapeutic strategy for advanced LUAD patients, particularly those with low THUMPD1 expression.

## 4 Conclusion

In summary, we have delineated a novel tumor‐suppressive signaling cascade in which THUMPD1 acts through IGF2R to inhibit AKT signaling, thereby relieving the repression on AMPK and leading to its activation. The subsequent AMPK‐driven upregulation of SLC31A1 leads to intracellular Cu^+^ accumulation and ultimately triggers cuproptosis accompanied by lethal mitophagy. This work not only deepens our understanding of LUAD pathogenesis by revealing the sophisticated interplay between epitranscriptomic regulation and metabolic cell death but also provides a solid rationale and compelling pre‐clinical evidence for repositioning elesclomol as a targeted therapy for LUAD.

## 5 Experimental Section

### 5.1 Clinical Samples

Human LUAD tissues were obtained from FAHZZU Biobank (Table S1). The use of human tissues was approved by the the Ethics Committee of Scientific Research and Clinical Trial of FAHZZU (2023‐KY‐0958‐002) and informed written consents were obtained from all participants. Tissue samples were immediately snap‐frozen in liquid nitrogen after resection and stored at −80°C until protein or RNA extraction. For tumor microarrays (TMAs), formalin‐fixed, paraffin‐embedded tissue blocks were procured from Shanghai OUTDO Biotech Co., Ltd. (Shanghai, China). The TMA comprised 56 samples (Table S2). Sections were immunostained with a primary antibody, and IHC scores were quantitatively assessed.

### 5.2 Cell Culture and Stable Cell Line Generation

Human LUAD cell lines A549 (RRID: CVCL_0023), PC‐9 (RRID: CVCL_B260), and H1975 (RRID: CVCL_1511) were obtained from Procell Life Science & Technology Co., Ltd (Wuhan, China). All cell lines were regularly authenticated through Short Tandem Repeat profiling and confirmed to be free of mycoplasma contamination using a PCR‐based detection kit. Cells were cultured in RPMI‐1640 medium supplemented with 10% fetal bovine serum at 37°C in a humidified atmosphere with 5% CO2. For in vitro experiments, cells were additionally treated with Z‐VAD‐FMK (20 µM, MCE, HY‐16658B), tetrathiomolybdate (TTM, 10 µM, MCE, HY‐100717), SC79 (1 nM, MCE, HY‐18749), bafilomycin A1 (BafA1, 100 nM, MCE, HY‐100558), or staurosporine (STS, 1 µM, MCE, HY‐15141), as indicated in the corresponding experiments. In experiments involving copper‐dependent cell death induction, elesclomol (ES) (MCE, HY‐12040) was combined with CuCl_2_ at a 1:1 ratio with indicated different concentrations for 72 h. For gene perturbation, lentiviral vectors were obtained from Genechem Co., Ltd (Shanghai, China). The sequences of shRNAs are listed in Table S3.

### 5.3 Animal Models

All animal procedures were conducted in compliance with the Animal Research: Reporting of In Vivo Experiments (ARRIVE) guidelines and were approved by the Ethics Committee of Scientific Research and Clinical Trial of FAHZZU (2023‐KY‐0958‐002). The study protocols adhered to the committee's criteria for humane endpoints, which stipulated that tumor burden should not exceed 10% of the animal's initial body weight or a mean tumor diameter of 20 mm.

To evaluate tumor growth and metastasis, subcutaneous xenograft and experimental metastasis models were established. Briefly, 100 µL suspensions containing 1 × 10^6^ A549 or PC‐9 cells, which were stably transduced with either shTHUMPD1 (knockdown) or THUMPD1‐OE (overexpression) constructs, were injected into the flanks or via the tail vein of 4‐6‐week‐old female BALB/c nude mice, respectively. Tumor volume in the subcutaneous model was measured every three days. All mice were euthanized 3–6 weeks post‐injection. Lung tissues from the tail vein injection groups were harvested for the quantification of metastatic nodules and subsequent analysis.

For the in vivo drug treatment, TTM was administered intraperitoneally at 1 mg/kg, five times per week, starting 7 days after tumor‐cell injection. For the THUMPD1‐knockdown rescue experiments, ES was administered intraperitoneally at 10 mg/kg every 2 days, starting 7 days after tumor‐cell injection. For in vivo AKT reactivation experiments, SC79 was administered intraperitoneally at 20 mg/kg every 3 days, starting 7 days after tumor‐cell injection.

To investigate the role of THUMPD1 in a genetically engineered and chemically induced model of LUAD, whole‐body *Thumpd1*‐knockout (KO) mice on a C57BL/6N background were generated by CRISPR/Cas9‐mediated deletion of exon 2 of the *Thumpd1* gene. Viable adult KO mice were used for urethane‐induced LUAD experiments beginning at 6–8 weeks of age. LUAD was induced by weekly intraperitoneal injections of urethane (1 g/kg) for 10 months. Starting from the fourth month, KO mice were randomly allocated into two treatment groups receiving either elesclomol (10 mg/kg, i.p.) or phosphate‐buffered saline twice per week. Following the treatment period, lung tissues were collected, and the tumor burden was assessed by counting the number of pulmonary nodules.

### 5.4 Silver Staining and Mass Spectroscopy (MS)

Protein extracts of equal quantity, obtained from homogenized tissues, were resolved by 10% SDS‐PAGE. The gel was then subjected to silver staining using a Fast Silver Stain Kit (Beyotime, P0017s) in accordance with the manufacturer's protocol. Briefly, the gel was fixed overnight in a solution of 50% ethanol, 10% acetic acid, and 40% water at room temperature with gentle shaking. Following fixation, the gel was sequentially washed with 30% ethanol and deionized water. Protein bands were subsequently visualized, and those of interest were excised for further analysis by LC‐MS/MS, which was performed by Wininnovate Bio‐Tech Co. (Shenzhen).

### 5.5 Acquisition of LUAD Patient Datasets

The gene expression profiles and corresponding clinical data for LUAD patients were obtained from 11 independent cohorts (GSE26939, GSE30219, GSE72094, GSE50081, GSE31210, GSE68465, GSE14814, GSE41271, GSE37745, and GSE27716) in the Gene Expression Omnibus (GEO) database (https://www.ncbi.nlm.nih.gov/geo/). Additionally, the prognostic value of genes of interest was assessed using the Kaplan‐Meier plotter online database [48].

### 5.6 RNA Extraction and Quantitative Real‐Time PCR (qRT‐PCR)

Total RNA was extracted using the HiPure RNA Plus Mini Kit (Magen Biotechnology Co., Ltd., Guangzhou, China), which enriches for mRNA during the purification process. The extracted RNA was then reverse transcribed into complementary DNA (cDNA) using the ReverTra Ace qPCR RT Master Mix (Toyobo, Japan). qRT‐PCR was performed on a CFX96 system (Bio‐Rad) with SYBR Green Real‐time PCR Master Mix (Toyobo). The primers used in the study are listed in Table S3.

### 5.7 Western Blot

Proteins were extracted from tissues and cells using RIPA lysis buffer containing protease and phosphatase inhibitors. Protein concentration was determined using a BSA assay. Equal amounts of protein were separated by SDS‐PAGE and transferred to NC membranes (Millipore). After blocking with 5% non‐fat milk, membranes were incubated with primary antibodies overnight at 4°C, followed by incubation with HRP‐conjugated secondary antibodies. Protein bands were visualized using an enhanced chemiluminescence (ECL) detection system (Applygen, P1050). Antibodies against THUMPD1 (Proteintech, 14921‐1‐AP), anti‐puromycin (Abcam, ab315887), IGF2R (Proteintech, 20253‐1‐AP), SLC31A1 (ABclonal, A10109), FDX1 (Proteintech, 12592‐1‐AP), Lipoic acid antibody (Abcam, ab58724), PINK1 (Abcam, ab216144), Parkin (Servicebio, GB15596), LC3 (Abmart, T55992), p‐AKT (Abmart, T40067), AKT (Abmart, T55561), p‐AMPK (CST, #2535), AMPK (CST, #5831), PPP2R1A (HUABIO, HA500160) and β‐actin (Servicebio, GB15003) were used.

### 5.8 Dot Blot

RNA samples were denatured and spotted onto a positively charged nylon membrane (Servicebio, G6018). The membrane was UV cross‐linked, blocked with 5% non‐fat milk, and incubated with an anti‐ac4C antibody (Proteintech, 68498‐1‐Ig) overnight at 4°C. After washing, the membrane was incubated with an HRP‐conjugated secondary antibody and detected by ECL. Methylene blue staining (Solarbio, M8030) was used to confirm equal RNA loading.

### 5.9 ac4C Quantification by LC‐MS/MS

The quantification of ac4C in RNA was performed by liquid chromatography‐tandem mass spectrometry (LC‐ESI‐MS/MS). Briefly, 1 µg of total RNA was enzymatically digested into individual nucleosides using a cocktail of S1 nuclease, alkaline phosphatase, and phosphodiesterase I. The resulting digest was then extracted with chloroform. The aqueous phase containing the nucleosides was analyzed using a UPLC‐ESI‐MS/MS system equipped with a HSS T3 C18 column. Separation was achieved using a methanol‐water gradient mobile phase, and detection was carried out in positive ion multiple reaction monitoring (MRM) mode on a QTRAP 6500+ mass spectrometer for specific and sensitive quantification.

### 5.10 Polysome Profiling

Polysome profiling was performed using an established protocol with modifications provided by Hangzhou NeoRibo Biotechnology Co., Ltd. Briefly, cells were lysed under conditions that preserve polysomes, and the clarified lysates were layered onto a 10%–50% (w/v) linear sucrose gradient. Following ultracentrifugation in an SW41 rotor at 38,000 rpm for 3 h at 4°C, the gradients were fractionated using a density gradient fractionation system with continuous monitoring of absorbance at 254 nm. Fractions corresponding to monosomes and polysomes were collected for RNA isolation and subsequent analysis.

### 5.11 Translation Efficiency Analysis

Cells were incubated with puromycin (1 µM) for 30 min. Proteins were then extracted and subjected to western blot using the anti‐puromycin antibody to assess nascent protein synthesis.

### 5.12 mRNA‐seq

Total RNA was extracted using TRIzol Reagent (Thermo Fisher) and treated with DNase I (NEB) to remove genomic DNA. RNA quality was assessed by measuring A260/A280 ratio (Nanodrop), evaluating integrity (LabChip GX Touch), and quantifying concentration (Qubit 3.0). Sequencing libraries were constructed from the qualified RNA using the KCTM Digital mRNA Library Prep Kit (Seqhealth Tech), which incorporates 12‐bp unique molecular identifiers (UMIs) to correct for PCR duplication bias. The final libraries (200‐500 bp inserts) were sequenced on a DNBSEQ‐T7 platform (MGI) in PE150 mode. UID mRNA‐seq experiment, library preparation, high through‐put sequencing and data analysis were conducted by Seqhealth Technology Co., Ltd, Wuhan, China.

### 5.13 Acetylated RNA Immunoprecipitation (acRIP)‐seq

The enriched polyA+ RNA was fragmented to ∼100 nucleotides. A portion of the fragmented RNA was saved as input control, while the remainder was subjected to immunoprecipitation using an anti‐ac4C monoclonal antibody (Abcam, 252215) and protein G magnetic beads. After washing, the immunoprecipitated RNA was extracted. Libraries for both input and IP samples were constructed using the KCTM Digital mRNA Library Prep Kit (Seqhealth Tech), which employs unique molecular identifiers (UMIs) to eliminate PCR duplication bias. The final libraries, sized between 200–500 bp, were sequenced on a DNBSEQ‐T7 platform (MGI) in PE150 mode. UID ac4C acRIP‐seq experiment, library preparation, high through‐put sequencing and data analysis were conducted by Seqhealth Technology Co., Ltd, Wuhan, China.

### 5.14 RNA Immunoprecipitation (RIP)‐seq

Approximately 1 × 10^7^ cells were washed twice with ice‐cold PBS and lysed on ice. Ten percent of the lysate was reserved as Input; 80% was incubated with a specific anti‐THUMPD1 antibody (Proteintech, 14921‐1‐AP), and 10% with rabbit IgG (Abcam, ab171870) as a negative control (IgG). After stringent washes, immune complexes were eluted and RNA was extracted with TRIzol (Thermo Fisher, 15596018CN), quantified on a Qubit 3 fluorometer, and assessed for integrity by agarose gel electrophoresis. Immunoprecipitation efficiency was verified by WB. Strand‐specific libraries were prepared using the KCTM Digital Stranded mRNA Library Prep Kit (Seqhealth), which incorporates 12‐base unique molecular identifier (UMI) to reduce PCR/sequencing duplication, size‐selected for ∼200–500 bp, quantified, and sequenced on a DNBSEQ‐T7 (MGI) platform with PE150 reads. RIP experiment, high through‐put sequencing and data analysis were conducted by Seqhealth Technology Co., LTD (Wuhan, China).

### 5.15 Ribosome (Ribo)‐seq

For Ribo‐seq library preparation, 1×107 cells were lysed in a buffer containing 100 µg/mL cycloheximide to arrest ribosomes. The lysate was treated with RNase I to digest unprotected RNA, and ribosome‐protected fragments (RPFs) were then isolated by size exclusion chromatography. RPFs ranging from 26 to 32 nt were purified by PAGE gel extraction, followed by ribosomal RNA depletion. Sequencing libraries were constructed using the QIAseq miRNA Library Kit (Qiagen) and subjected to paired‐end 150 bp sequencing on an Illumina Novaseq X Plus platform. Ribo‐seq experiment and high through‐put sequencing and data analysis were conducted by Seqhealth Technology Co., Ltd., Wuhan, China.

### 5.16 RNA Pull‐Down Assay

Biotin‐labeled sense (target) and antisense (control) IGF2R RNA probes (Table S3) were individually incubated with cell lysates under gentle rotation at 4°C. After incubation, RNA–protein complexes were captured using streptavidin magnetic beads (Sigma‐Aldrich, S1638) according to the manufacturer's instructions. The beads were then washed extensively to remove nonspecific binding. Bound proteins were eluted by boiling in SDS loading buffer and subsequently analyzed by Western blotting with the indicated antibodies.

### 5.17 Co‐Immunoprecipitation (Co‐IP) and Immunoprecipitation‐Mass Spectrometry (IP‐MS)

Cells were lysed in IP lysis buffer supplemented with protease and phosphatase inhibitors. After clarification by centrifugation, equal amounts of protein lysates were pre‐cleared and then incubated overnight at 4°C with IGF2R monoclonal antibody (Invitrogen, MA1‐066) or a mouse IgG antibody as a negative control (Proteintech, B900620). Immune complexes were captured using protein A+G magnetic beads (Beyotime, P2108) according to the manufacturer's instructions. The beads were washed extensively with TBS. For Co‐IP validation, bound proteins were eluted by boiling in SDS loading buffer and analyzed by Western blotting with the indicated antibodies. For IP‐MS analysis, the bead‐bound immunoprecipitated complexes were submitted to Majorbio Bio‐Pharm Technology Co., Ltd. for mass spectrometry analysis and protein identification. The identified proteins were subsequently subjected to KEGG enrichment analysis.

### 5.18 Cell Proliferation and Colony Formation

Cell proliferation was assessed using the Cell Counting Kit‐8 (CCK‐8) (UElandy, C6005) according to the manufacturer's protocol. For colony formation assays, cells were seeded in 6‐well plates at a density of 1000 cells per well and cultured for 10 days. Colonies were fixed, stained with crystal violet, and counted.

### 5.19 Cell Migration and Invasion Assays

Cell migration and invasion abilities were assessed using the wound healing and Transwell assays, respectively. For the wound healing assay, cells were seeded in 6‐well plates and cultured until full confluence. A sterile 10 µL pipette tip was used to create a straight scratch in the monolayer. The initial gap width (w1) was recorded at 0 h, and the closure was measured after 24 h (w2). The relative migration rate was calculated as (w1 – w2) / w1 × 100%.

For the Transwell invasion assay, cell migratory and invasive capabilities were evaluated. For the migration assay, uncoated Transwell chambers (24‐well, Costar, Cambridge, MA) were used. For the invasion assay, the chambers were pre‐coated with Matrigel to simulate the extracellular matrix barrier. In both assays, homogeneous single‐cell suspensions were seeded into the upper chambers. After 24 h of incubation, non‐migratory or non‐invasive cells on the upper surface were carefully removed with a cotton swab. Cells that had migrated or invaded to the lower surface were fixed, stained with crystal violet, and imaged. The number of cells in five random fields per well was quantified using ImageJ software.

### 5.20 Flow Cytometric Analysis of Cell Death

Cell death was assessed using the APC‐Annexin V/PI apoptosis detection kit (UElandy, A6030) according to the manufacturer's instructions. Briefly, cells were digested with trypsin without EDTA, and both floating and attached cells were collected to avoid underestimation of cell death. After centrifugation, 1 × 10^6^ cells were resuspended in 100 µL 1× Annexin V binding buffer, followed by addition of 5 µL APC‐Annexin V and 5 µL PI. Samples were incubated for 15 min at room temperature in the dark, then diluted with 400 µL 1× Annexin V binding buffer and analyzed by flow cytometry.

### 5.21 Transmission Electron Microscopy (TEM)

Cells were prepared for TEM analysis as follows. Briefly, cell pellets were obtained by centrifugation and fixed in TEM fixative at 4°C. After washing with 0.1 m phosphate buffer (PB, pH 7.4), the cell pellets were pre‐embedded in 1% agarose to facilitate handling and post‐fixed with 1% OsO4 in PB. Dehydration was performed using a graded ethanol series followed by acetone. The samples were then infiltrated and embedded in EMBed 812 resin, which was polymerized at 60°C for 48 h. Ultrathin sections (60–80 nm) were cut and collected on copper grids. The sections were stained with uranyl acetate and lead citrate prior to observation and imaging under TEM.

### 5.22 Fluorescence In Situ Hybridization (FISH)

FISH was performed on paraffin‐embedded cells using SweAMI technology to detect IGF2R mRNA. Briefly, cells were fixed, dehydrated, and embedded in paraffin. After dewaxing and dehydration, sections underwent epitope retrieval and proteinase K digestion. Hybridization with IGF2R‐specific probes was carried out overnight at 40°C, followed by stringent washes. Signal amplification was achieved through sequential hybridization with branched and CY3‐labeled probes, generating a red signal for IGF2R mRNA. Nuclei were counterstained with DAPI. Fluorescent signals were visualized using a fluorescence microscope with standard filter sets for DAPI and CY3 (IGF2R mRNA). All steps were conducted under RNase‐free conditions.

### 5.23 Immunohistochemistry

Immunohistochemistry was performed on paraffin‐embedded mouse tumor and lung tissue sections. Briefly, sections were deparaffinized, rehydrated, and subjected to antigen retrieval under conditions optimized for the specific tissue and target antigen. Following peroxidase blocking with 3% H_2_O_2_ and nonspecific site blocking with 3% bovine serum albumin (BSA), the sections were incubated overnight at 4°C with the indicated primary antibodies, including THUMPD1 (Proteintech, 14921‐1‐AP), IGF2R (Proteintech, 20253‐1‐AP), and p‐AKT (Abmart, T40067). After washing, a corresponding horseradish peroxidase (HRP)‐labeled secondary antibody was applied. Signals were developed using a DAB substrate, and the reaction was monitored microscopically. Nuclei were counterstained with hematoxylin. Finally, sections were dehydrated, cleared in xylene, and mounted with a permanent medium for observation under a light microscope.

### 5.24 Mitochondrial Function Assays

Intracellular reactive oxygen species (ROS) and mitochondrial membrane potential (ΔΨm) were measured using commercial kits from Beyotime (ROS: S0033; JC‐1: C2003) according to the manufacturer's instructions. Signals were acquired by both fluorescence microscopy and flow cytometry. For JC‐1 staining, mitochondrial depolarization was reflected by a decreased red/green fluorescence ratio.

### 5.25 CopperSensor‐1

Cells were stained with CopperSensor‐1 (CS1; MedChemExpress, HY‐141511) according to the manufacturer's instructions, followed by fluorescence microscopy imaging.

### 5.26 Central Carbon Metabolite Profiling

Central carbon metabolite analysis was performed using high‐performance ion chromatography coupled with tandem mass spectrometry (HPIC‐MS/MS). Metabolites were extracted from samples through freeze‐thaw cycles and methanol precipitation, followed by analysis on a Thermo Scientific Dionex ICS‐6000 system with MRM detection. Method validation confirmed the reliability of quantification through established calibration curves, with LLOQ defined at S/N ≥ 10.

### 5.27 Statistical Analysis

Data are presented as the mean ± standard deviation (SD) from a minimum of three independent experiments. Statistical analyses were performed using GraphPad Prism (version 9.5, La Jolla, USA) and R (version 4.4.1). For comparisons between two groups, an unpaired two‐tailed Student's T‐test or the Wilcoxon test was applied, as appropriate. Comparisons among multiple groups were analyzed by one‐way ANOVA or Kruskal–Wallis test followed by an appropriate post‐hoc test. Survival curves were plotted using the Kaplan‐Meier method and compared with the log‐rank test. Univariate and multivariate Cox proportional‐hazards regression models were employed to assess the prognostic value of variables. Correlations between continuous variables were evaluated using Pearson's correlation coefficient. A p‐value < 0.05 was considered statistically significant.

### 5.28 Study Approval

This study was approved by the Ethics Committee of the First Affiliated Hospital with Zhengzhou University (2023‐KY‐0958‐002).

## Funding

Young and Middle‐Aged Health Science and Technology Innovation Outstanding Youth Talent Program of Henan Province (YXKC2022060); Cultivation Project of Henan Health Science and Technology Innovation Talents (YQRC2023011); Key Research Project of Higher Education in Henan Province (26A320042)

## Conflicts of Interest

The authors declare no conflict of interest.

## Author Contributions

P.Z. and Z.G. were responsible for data acquisition, data analysis, and drafting the manuscript. P.Z., W.D., and X.C. performed the original in vitro and in vivo experiments. During the major revision, K.W. and B.Q. were responsible for planning and designing the additional experiments; K.W. and B.Q. carried out the revision‐related experimental work, participated in data acquisition and analysis, and contributed to revising the manuscript and preparing the point‐by‐point response to the reviewers. K.Z., Y.Q., S.Y., and Z.G. provided advice on study design. P.Z., S.Z., X.L., and K.W. were responsible for the conception, design, and supervision of the study.

## Supporting information




**Supporting File 1**: advs75556‐sup‐0001‐SuppMat.docx.

**Supporting File 2**: advs75556‐sup‐0002‐TableS1.xlsx.

**Supporting File 3**: advs75556‐sup‐0003‐TableS2.xlsx.

**Supporting File 4**: advs75556‐sup‐0004‐TableS3.xlsx.

## Data Availability

The data supporting the findings of this study are publicly available in the NCBI Sequence Read Archive (SRA) under accession number PRJNA1338238 (https://www.ncbi.nlm.nih.gov/sra/?term=PRJNA1338238).
