# Supplementary material for: Cuproptosis and Mitophagy Mediated by the THUMPD1/IGF2R‐Dependent Suppression of AKT and Activation of AMPK Signaling Suppress Lung Adenocarcinoma Progression
Source: Adv Sci (Weinh). 2026 May 4;13(42):e21238. doi: 10.1002/advs.202521238 (PMC13335975; doi:10.1002/advs.202521238)
Supplement: Supplementary file 1 — Supporting File 1: advs75556‐sup‐0001‐SuppMat.docx. [file ADVS-13-e21238-s001.docx]

Supporting Information

Cuproptosis and mitophagy mediated by the THUMPD1/IGF2R-dependent suppression of AKT and activation of AMPK signaling suppress lung adenocarcinoma progression

Kai Wu, Bo Qin, Zhuoyu Gu, Weizheng Ding, Xiaoming Chen, Kaishang Zhang, Yujin Qiao, Shuang Yuan, Song Zhao, Xiangnan Li, Peng Zhang*


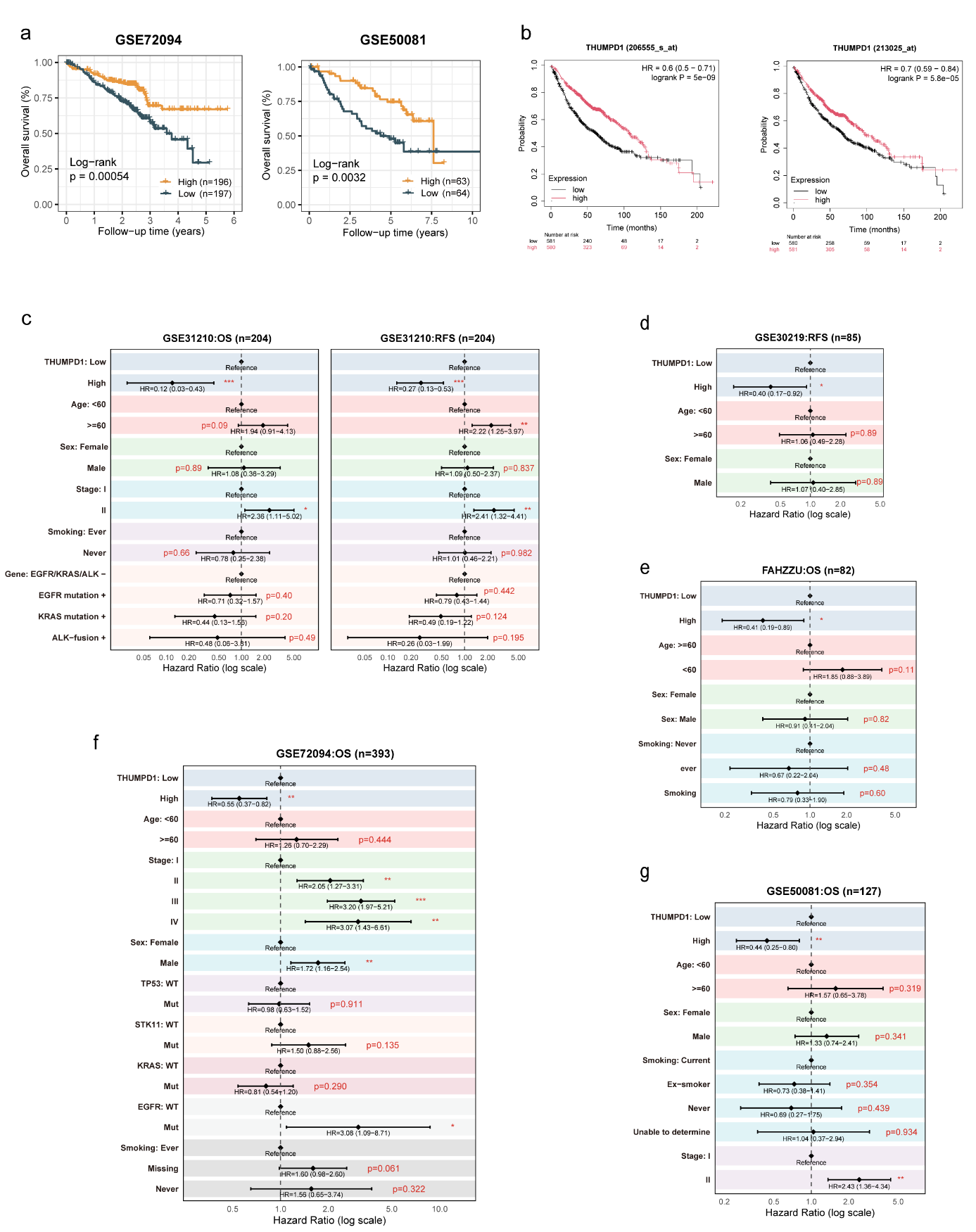


**Figure S1.** External survival validation of THUMPD1 in LUAD. a) Kaplan–Meier curves for overall survival (OS) in the GSE72094 cohort (n = 393) and the GSE50081 cohort (n = 127), stratified by THUMPD1 high versus low expression. b) Kaplan–Meier survival curves for two independent THUMPD1 probes in the KMplotter database. c) Multivariable Cox regression analysis for OS and recurrence-free survival (RFS) in the GSE31210 cohort (n = 204). d) Multivariable Cox regression analysis for RFS in the GSE30219 cohort (n = 85). e) Multivariable Cox regression analysis for OS in the FAHZZU cohort (n = 82). f) Multivariable Cox regression analysis for OS in the GSE72094 cohort (n = 393). g) Multivariable Cox regression analysis for OS in the GSE50081 cohort (n = 127). Log-rank tests were used in a and b. Multivariable Cox proportional hazards regression models were used in c–g. Significance: *p < 0.05; **p < 0.01; ***p < 0.001.


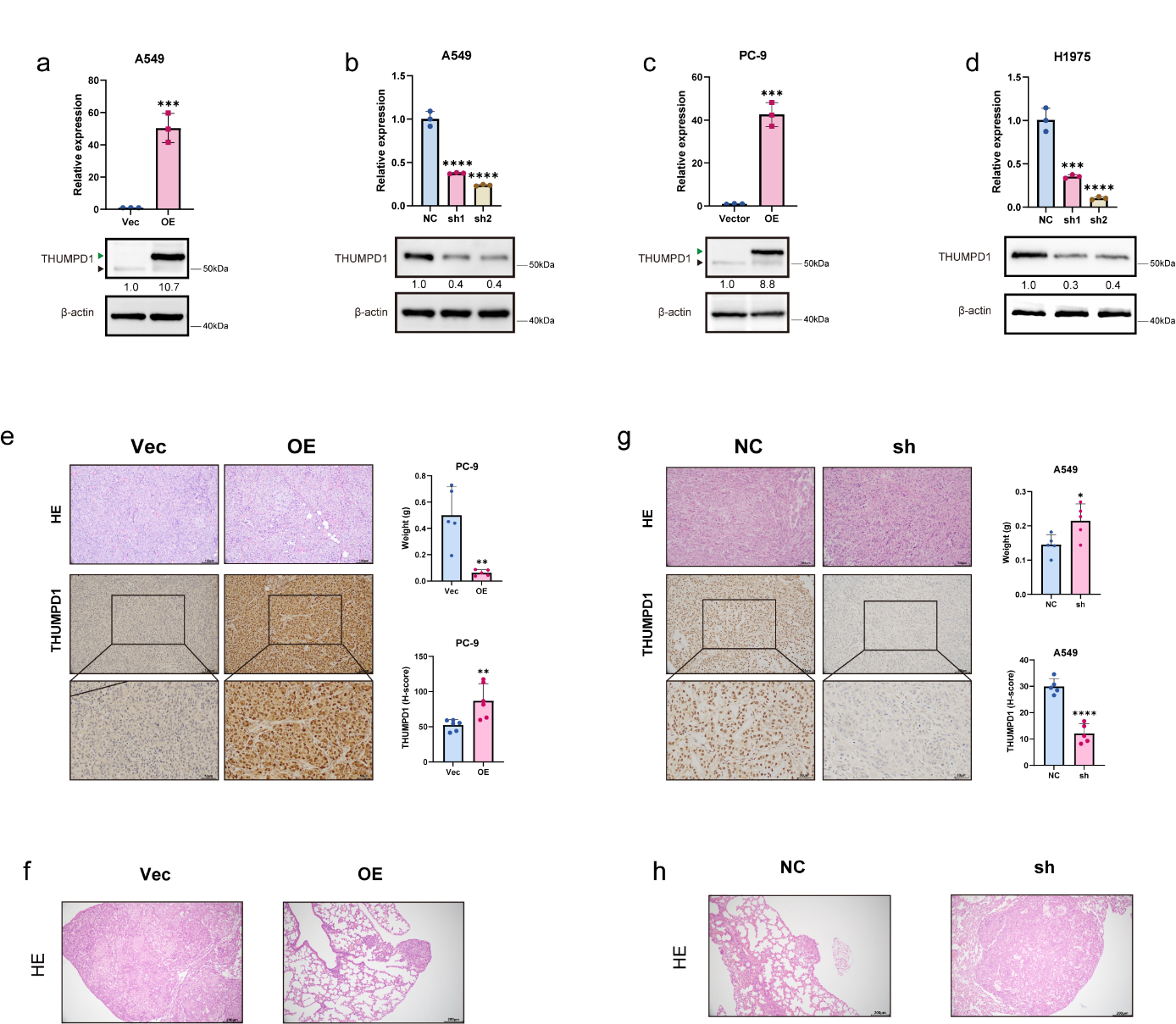


**Figure S2.** Validation of THUMPD1 perturbation and additional in vivo evidence for its tumor-suppressive role in LUAD. a–d) Validation of THUMPD1 perturbation in LUAD cells by qRT-PCR and Western blot, including THUMPD1 overexpression in A549 and PC-9 cells (a, c, green arrow: exogenous THUMPD1-Flag; black arrow: endogenous THUMPD1) and THUMPD1 knockdown in A549 and H1975 cells (b, d). e) Subcutaneous xenograft analysis using PC-9 cells with THUMPD1 overexpression, including representative haematoxylin and eosin (HE) staining, THUMPD1 immunohistochemistry (IHC), tumor weight quantification, and THUMPD1 H-score analysis. f) Representative HE images of lung metastatic lesions in the PC-9 tail-vein injection model. g) Subcutaneous xenograft analysis using A549 cells with THUMPD1 overexpression, including representative HE staining, THUMPD1 IHC, tumor weight quantification, and THUMPD1 H-score analysis. h) Representative HE images of lung metastatic lesions in the A549 tail-vein injection model. Values are mean ± s.d. of n = 3 biological replicates in a-d. Xenograft and metastasis experiments were performed with n = 5 mice per group in e–h. Two-tailed Student’s t-tests were used for comparisons between two groups, and one-way ANOVA was used for comparisons among multiple groups, as appropriate. Significance: *p < 0.05; **p < 0.01; ***p < 0.001; ****p < 0.0001. Original unprocessed blots are shown in Figure S11.


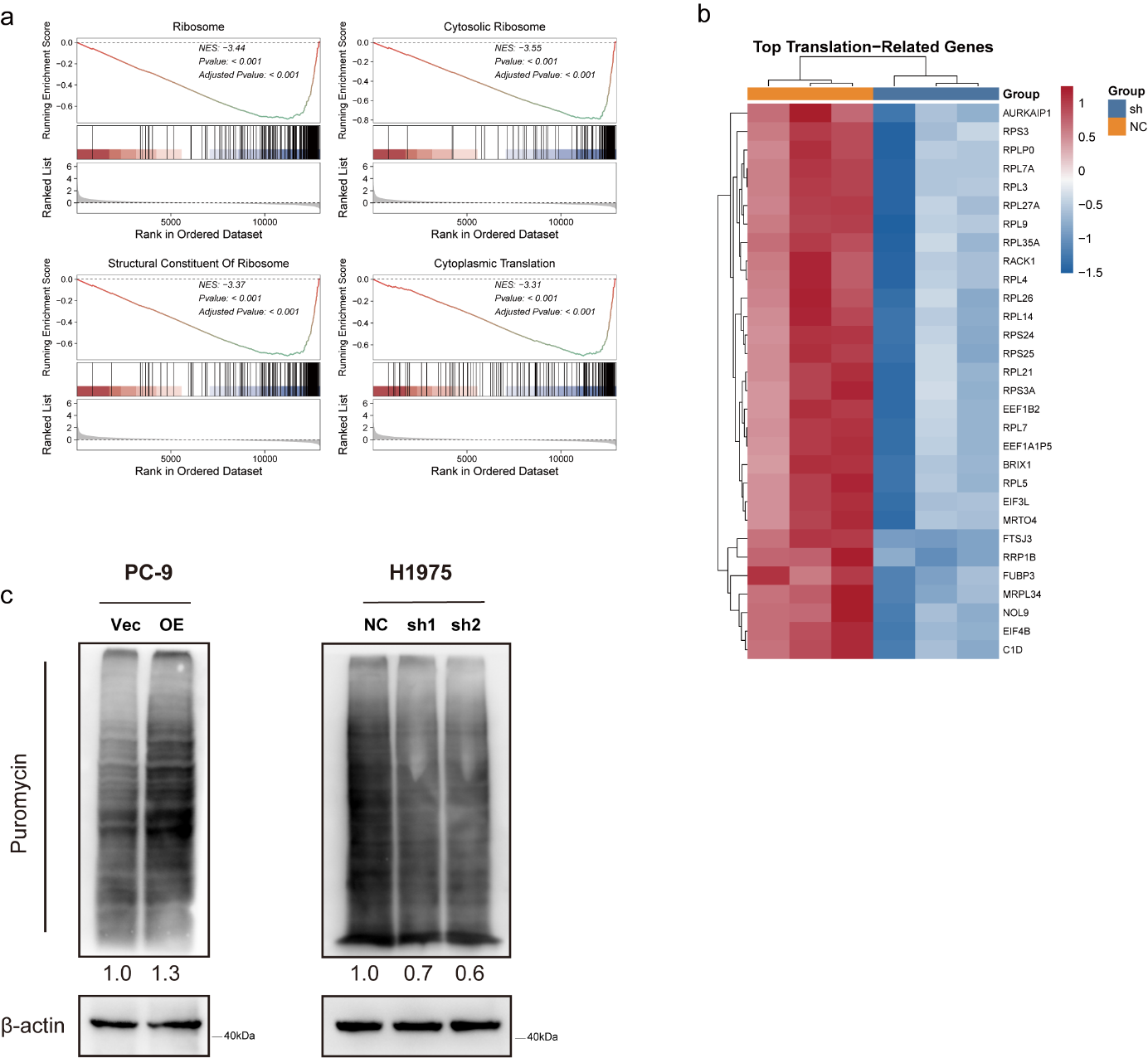


**Figure S3.** Additional evidence that THUMPD1 regulates translation-related pathways and global protein synthesis. a) Gene set enrichment analysis (GSEA) of mRNA-seq data showing downregulation of translation-related pathways in THUMPD1-knockdown cells. b) Heatmap showing representative translation-related genes downregulated upon THUMPD1 knockdown. c) Puromycin incorporation assays in PC-9 cells with THUMPD1 overexpression and H1975 cells with THUMPD1 knockdown (n = 3 biological replicates). Original unprocessed blots are shown in Figure S11.


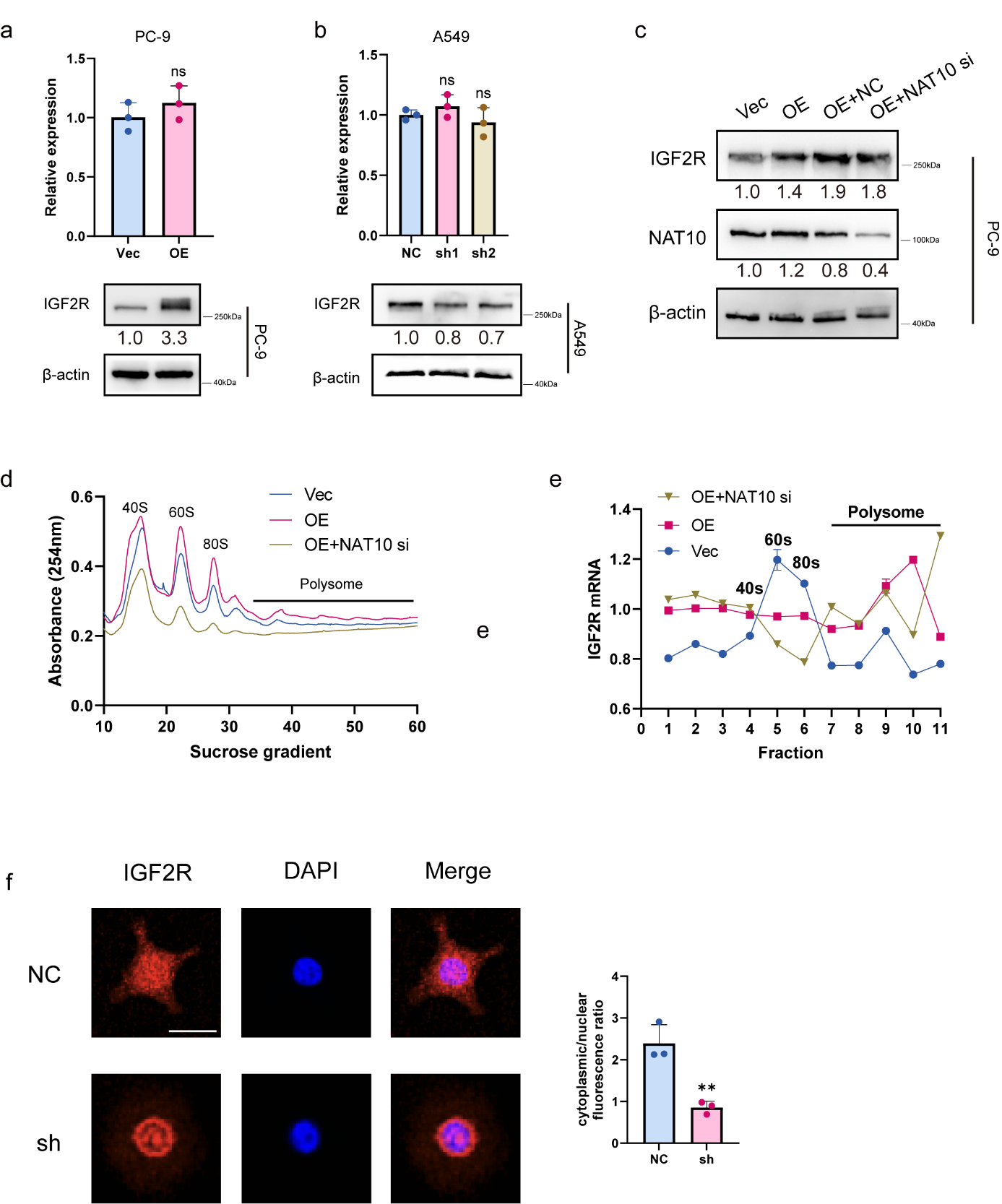


**Figure S4.** Additional validation that THUMPD1 regulates IGF2R expression independently of NAT10/ac4C. a-b) qRT-PCR and Western blot analyses of IGF2R expression in PC-9 (a) and A549 (b) cells. c) Western blot of IGF2R expression in PC-9 cells with THUMPD1 overexpression combined with NAT10 knockdown. d) Polysome profiling of the indicated groups. e) qRT-PCR analysis of IGF2R mRNA distribution across polysome fractions in the indicated groups. f) RNA fluorescence in situ hybridization (FISH) showing subcellular localization of IGF2R mRNA in THUMPD1-knockdown cells, with corresponding quantification. Values are mean ± s.d. of n = 3 biological replicates in a–c, e and f. Two-tailed Student’s t-tests were used for comparisons between two groups, and one-way ANOVA was used for comparisons among multiple groups, as appropriate. Significance: **p < 0.01; ns, not significant. Original unprocessed blots are shown in Figure S11.


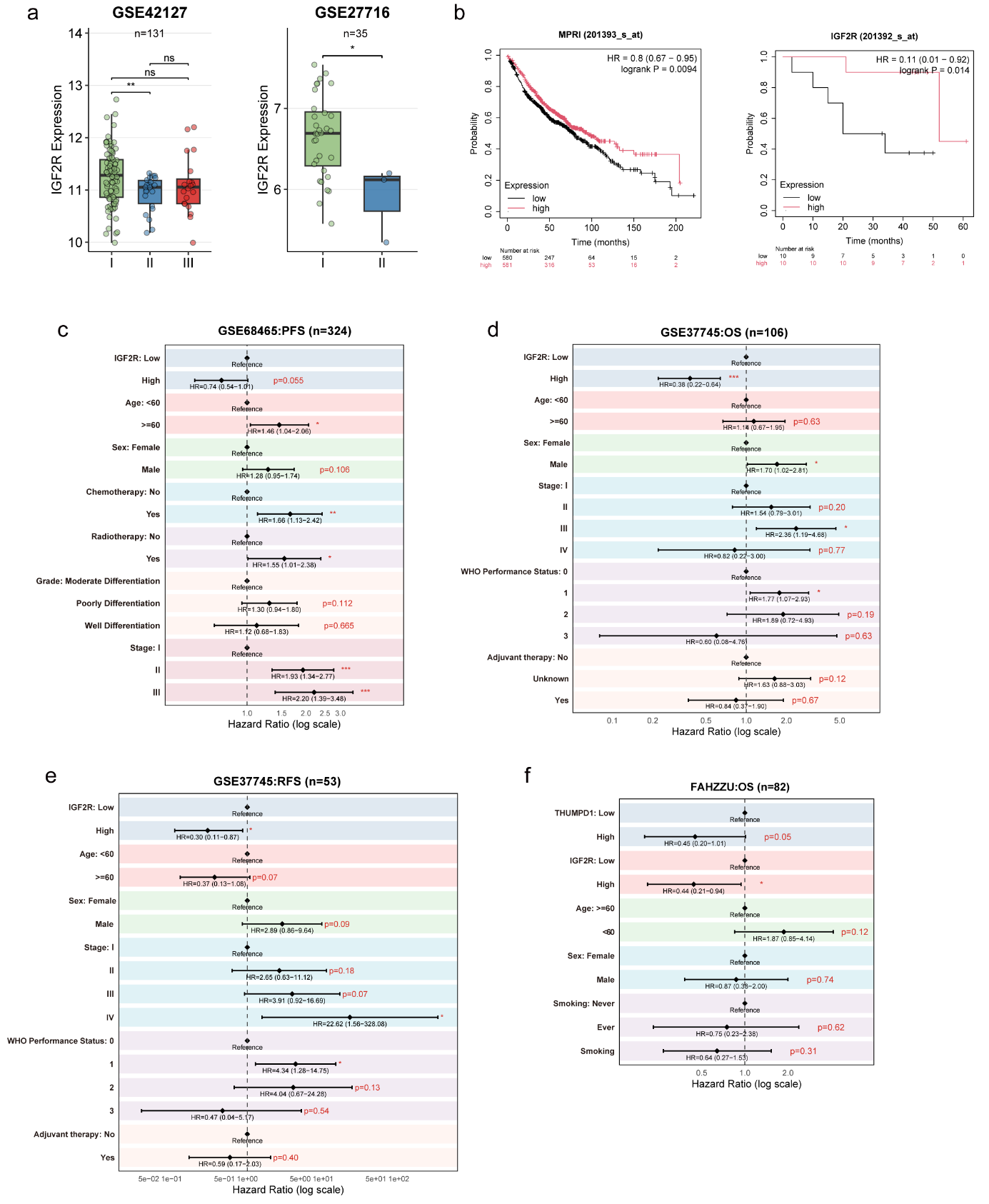


**Figure S5.** Clinical association and prognostic significance of IGF2R in independent LUAD cohorts. a) Boxplots showing IGF2R expression across AJCC stages in the GSE42127 (n = 131) and GSE27716 (n = 35) cohorts. b) Kaplan–Meier survival curves for different IGF2R probes in the KMplotter database. c) Multivariable Cox regression analysis of progression-free survival (PFS) for IGF2R in the GSE68465 cohort (n = 324). d) Multivariable Cox regression analysis of OS for IGF2R in the GSE37745 cohort (n = 106). e) Multivariable Cox regression analysis of RFS for IGF2R in the GSE37745 cohort (n = 53). f) Multivariable Cox regression analysis of OS in the FAHZZU cohort (n = 82), including both THUMPD1 and IGF2R. In a, a Kruskal–Wallis test was used for GSE42127 and a Wilcoxon test was used for GSE27716. Log-rank test was used in b. Multivariable Cox proportional hazards regression models were used in c–f. Significance: *p < 0.05; **p < 0.01; ***p < 0.001; ns, not significant.


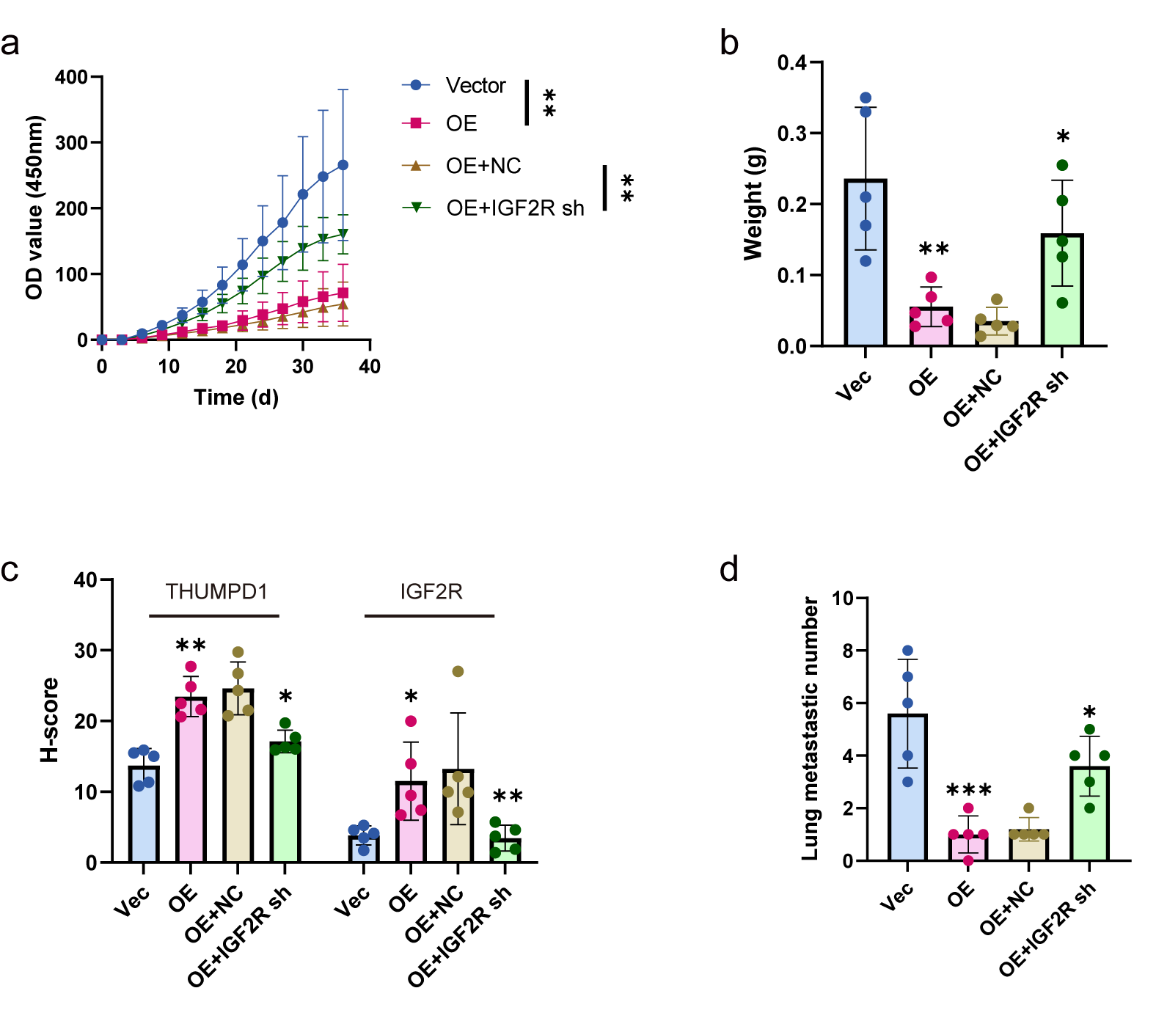


**Figure S6.** IGF2R mediates the tumor-suppressive effects of THUMPD1 in vivo. a) Subcutaneous xenograft growth curves in the indicated groups. b) Quantification of tumor weight in the indicated groups. c) H-score quantification of THUMPD1 and IGF2R IHC staining in xenograft tumors. d) Quantification of pulmonary metastatic nodules in the experimental lung-metastasis model. Values are mean ± s.d. of n = 5 mice per group. Comparisons among multiple groups were analyzed using one-way ANOVA. Significance: *p < 0.05; **p < 0.01; ***p < 0.001.


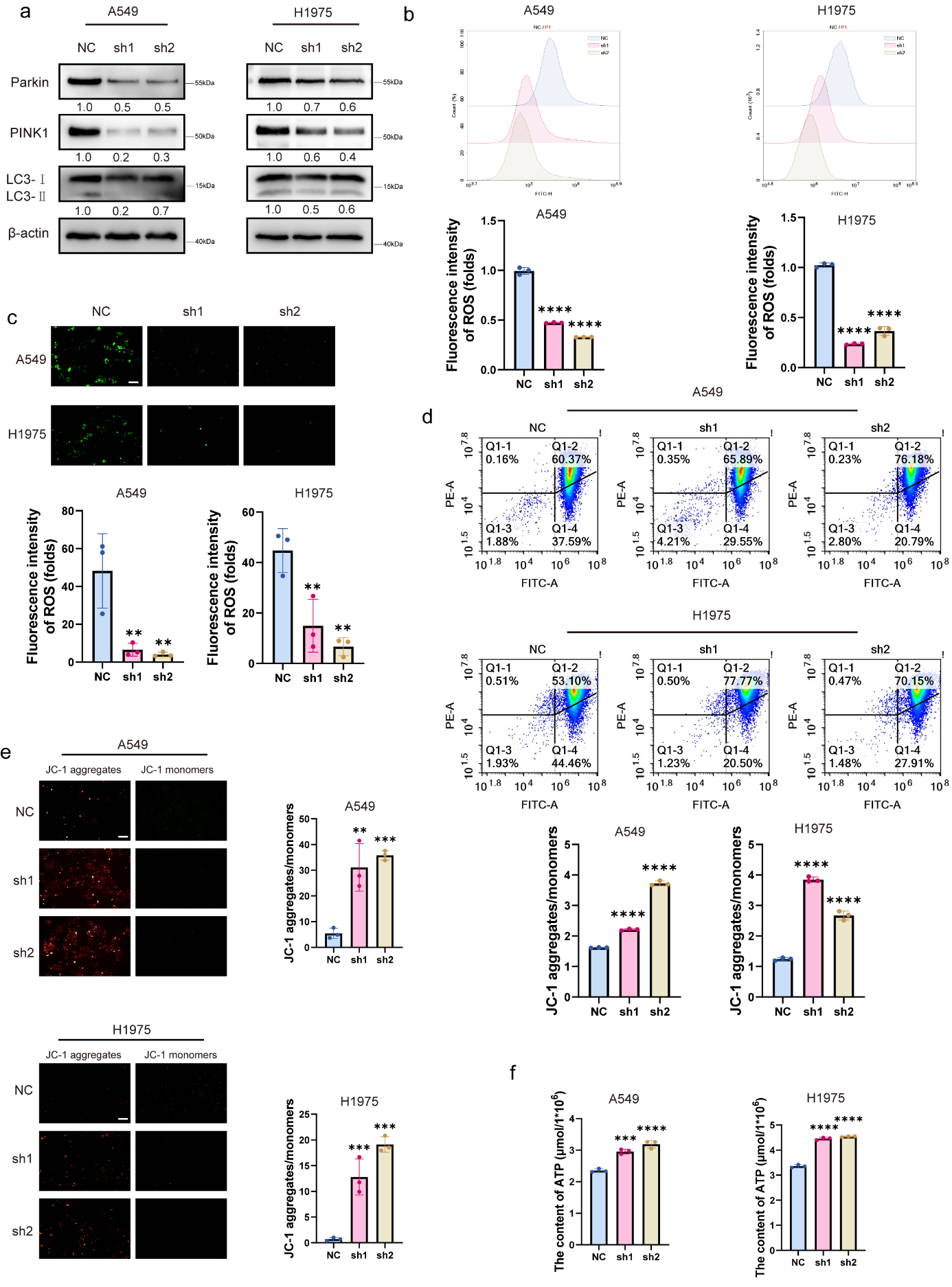


**Figure S7.** THUMPD1 knockdown confers resistance to mitophagy-related stress. a) Western blot of Parkin, PINK1, and LC3 (LC3-I/II) in A549 and H1975 THUMPD1 knockdown cells. b) Flow-cytometry histograms of intracellular ROS (DCFH-DA) with quantification. c) Representative DCF fluorescence images (scale bar: 200 μm) and corresponding intensity quantification. d) JC-1 flow-cytometry analysis of mitochondrial membrane potential and quantification. e) JC-1 microscopy (scale bar: 200 μm) showing aggregates (red)/monomers (green) and the quantification. f) Cellular ATP levels in A549 and H1975. Values are mean ± s.d. of n = 3 biological replicates. One-way ANOVA was used for comparisons among multiple groups. Significance: **p < 0.01; ***p < 0.001; ****p < 0.0001. Original unprocessed blots are shown in Figure S11.


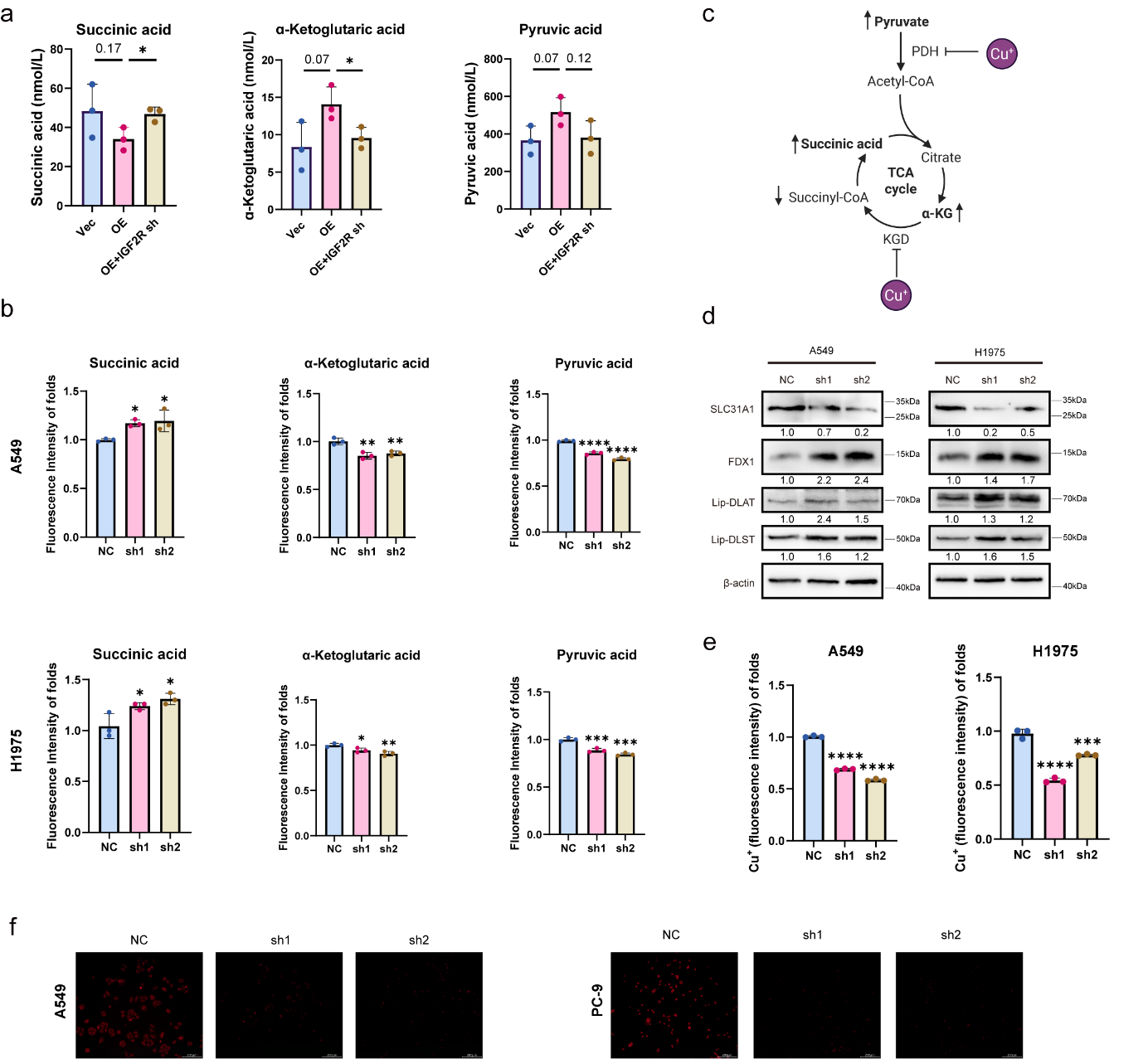


**Figure S8.** Metabolic and signaling profiles supporting the coupling of THUMPD1–IGF2R axis to cuproptosis and mitophagy. a) Bar plots of targeted central carbon metabolomic analysis showing metabolic alterations. b) Validation of central-carbon metabolites by enzymatic kits. c) Schematic of copper-induced disruption of lipoylated tricarboxylic acid (TCA) enzymes (PDH/KGDH) leading to the observed metabolite shifts. d) Western blot of SLC31A1, FDX1, and lipoylated DLAT/DLST under THUMPD1-knockdown conditions. e) Intracellular Cu⁺ levels under THUMPD1-knockdown conditions. f) Representative CopperSensor-1 (CS1) fluorescence images in A549 and H1975 cells with THUMPD1 knockdown. Values are mean ± s.d. of n = 3 biological replicates. A two-tailed Student’s t-test was used in a. One-way ANOVA was used in b and e. Significance: *p < 0.05; **p < 0.01; ***p < 0.001; ****p < 0.0001. Original unprocessed blots are shown in Figure S12.


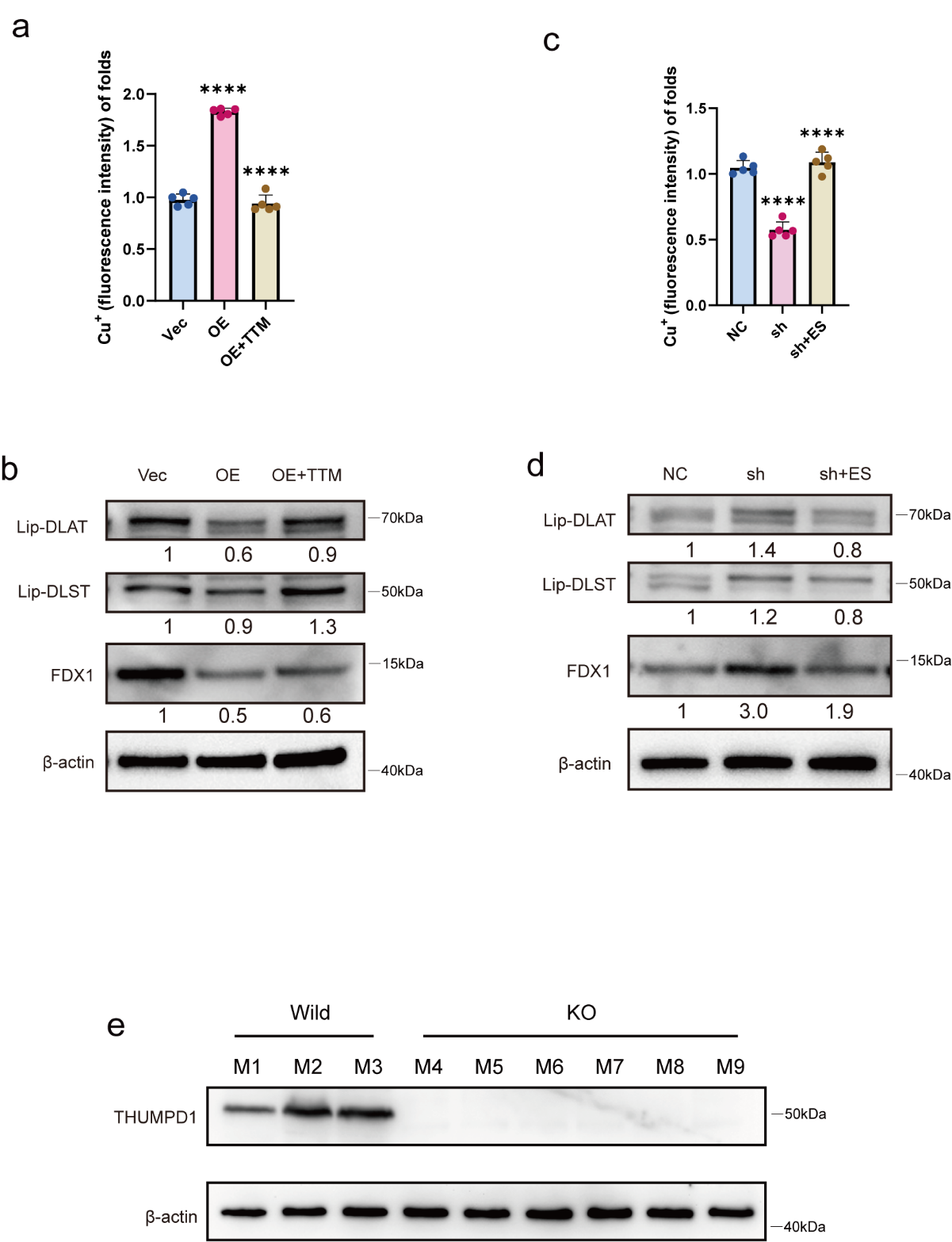


**Figure S9.** Intratumoral copper levels and signaling changes associated with copper-dependent tumor suppression. a) Intratumoral Cu⁺ levels in xenograft tumors from the tetrathiomolybdate (TTM) treatment groups. b) Western blot of lipoylated DLAT, DLST, and FDX1 in xenograft tumors from the TTM treatment groups. c) Intratumoral Cu⁺ levels in xenograft tumors from the elesclomol (ES) treatment groups. d) Western blot analysis of lipoylated DLAT, DLST, and FDX1 in xenograft tumors from the ES treatment groups. e) Western blot analysis of THUMPD1 in lung tissues from wild-type and *Thumpd1*-knockout (KO) mice. Values are mean ± s.d. of n = 5 biological replicates in a-d, and n = 3 biological replicates in e. One-way ANOVA was used for comparisons among multiple groups. Significance: ****p < 0.0001. Original unprocessed blots are shown in Figure S12.


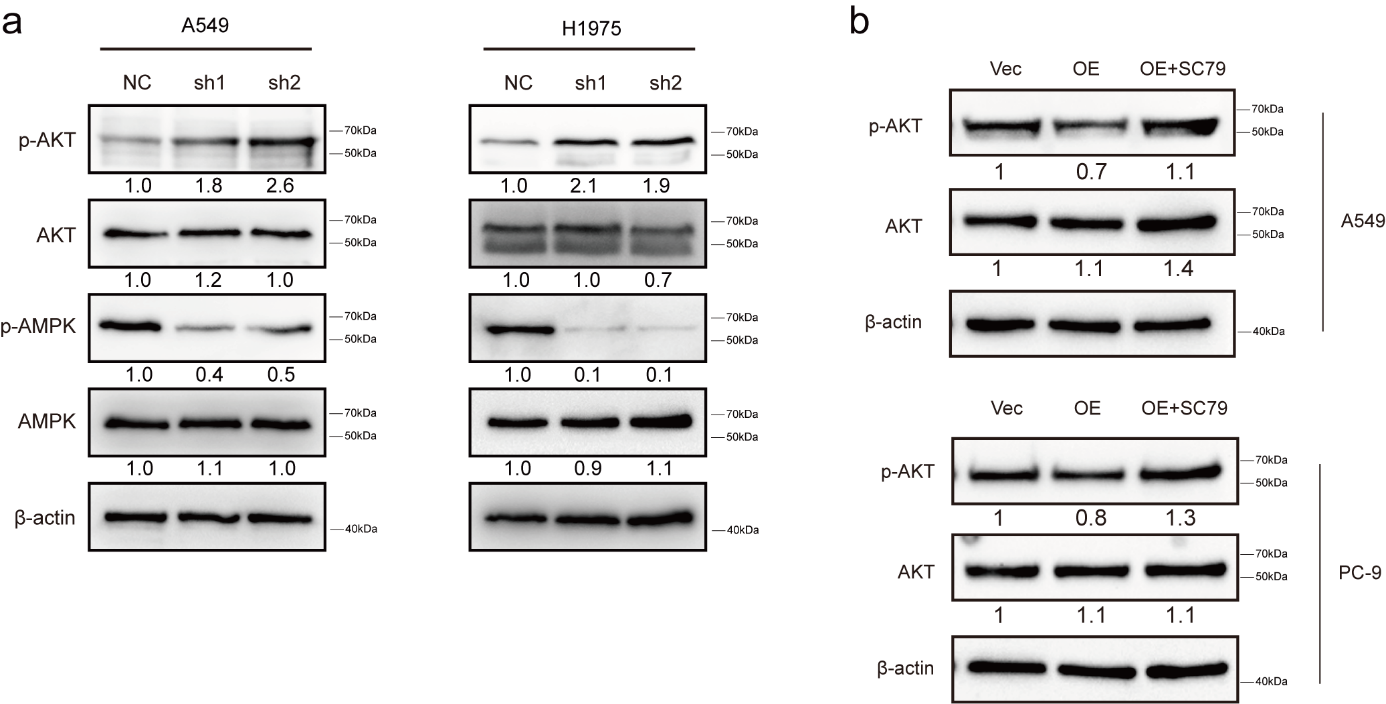


**Figure S10.** Additional validation of AKT/AMPK signaling changes downstream of THUMPD1. a) Western blot of AKT/AMPK pathway proteins in A549 and H1975 cells with THUMPD1 knockdown. b) Western blot of AKT pathway proteins in A549 and PC-9 cells with THUMPD1 overexpression following SC79 treatment. Blots are representative of n = 3 independent experiments. Original unprocessed blots are shown in Figure S12.


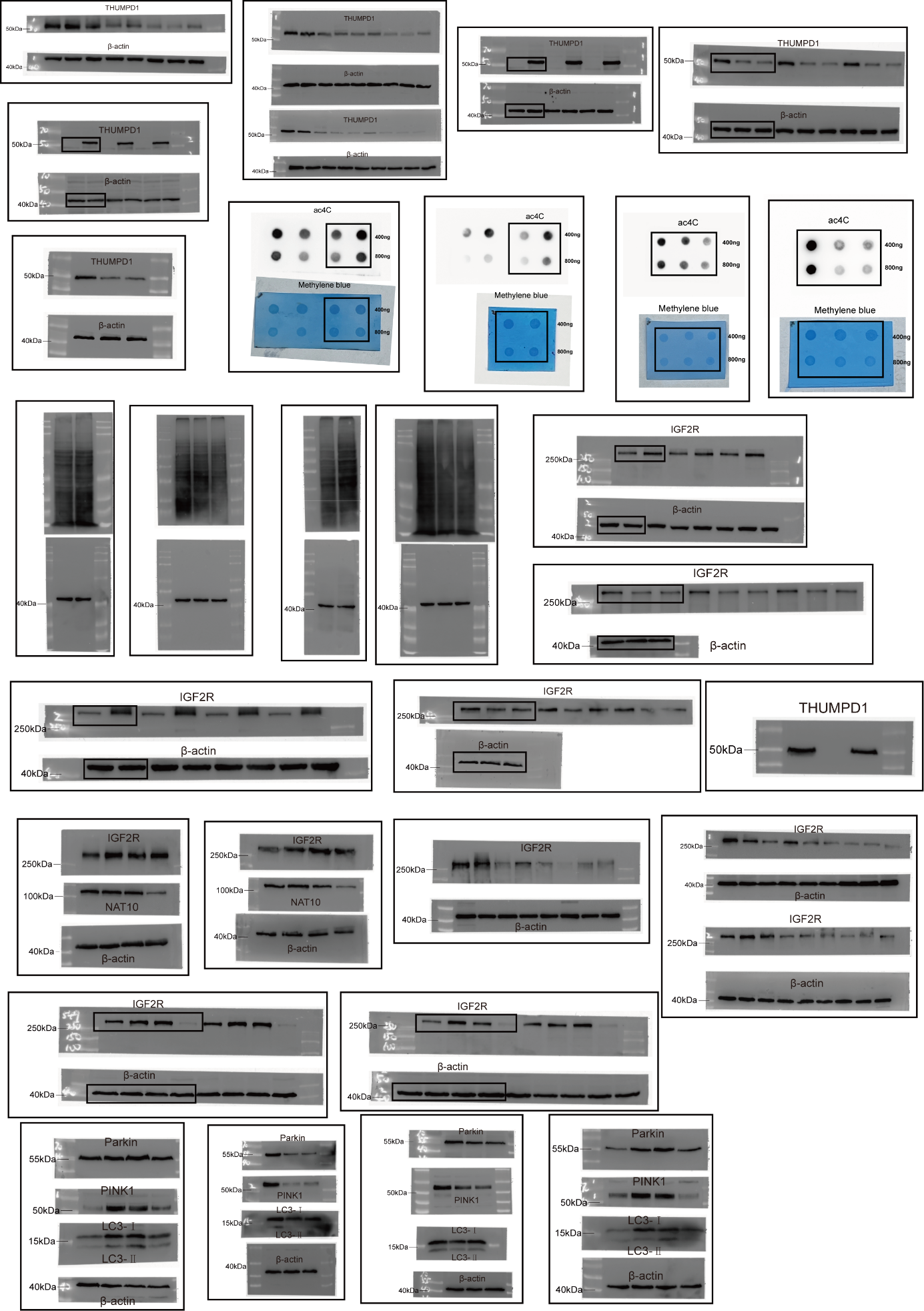


**Figure S11.** Original unprocessed blots corresponding to the main figures. Original unprocessed blots for the Western blot panels shown in Figures 1b,g, 3a–d,i, 4m,n,p,r, 5a,b, 6a, and 7b, as well as Figures S2a–d, S3c, S4a–c, and S7a.

**
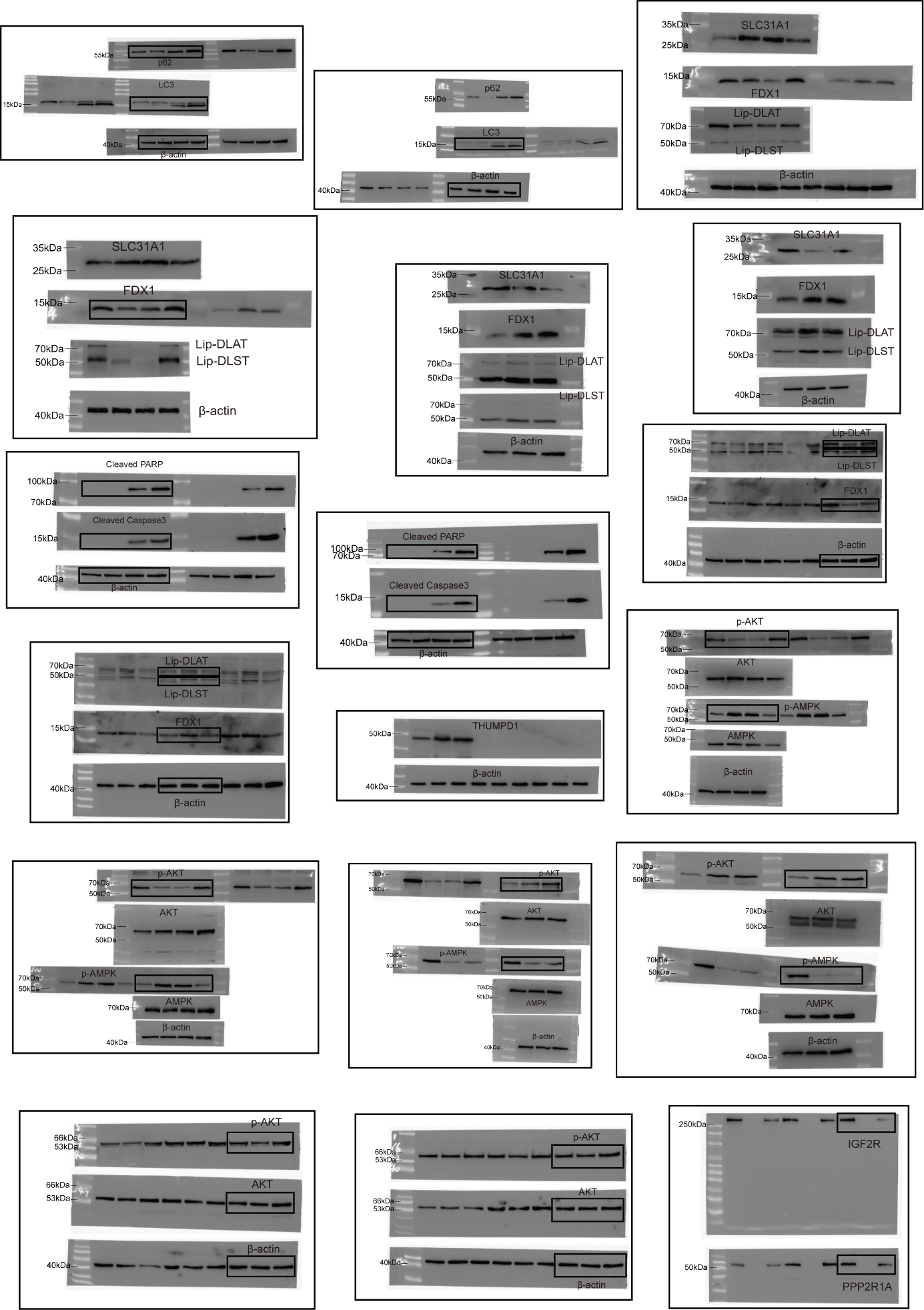
**

**Figure S12.** Additional original unprocessed blots corresponding to the indicated main and supplementary figures. Original unprocessed blots for the Western blot panels shown in Figures 7c, 8c, 9c, and 10a, as well as Figures S8d, S9b–e, and S10a,b.

**Table S1.** The mRNA expression levels of THUMPD1 and IGF2R and corresponding clinical information in 82 LUAD tissues.

**Table S2.** H-score of THUMPD1 and IGF2R and clinical information in TMA.

**Table S3**. Primers, sh and siRNAs and probes used in this study.
